# A Kinome RNAi Screen in *Drosophila* Identifies Novel Genes Interacting with Lgl, aPKC, and Crb Cell Polarity Genes in Epithelial Tissues

**DOI:** 10.1534/g3.117.043513

**Published:** 2017-06-13

**Authors:** Linda M. Parsons, Nicola A. Grzeschik, Kasun Amaratunga, Peter Burke, Leonie M. Quinn, Helena E. Richardson

**Affiliations:** * Cell Cycle and Development Laboratory, Research Division, Peter MacCallum Cancer Centre, East Melbourne, Victoria 3002, Australia; † Department of Anatomy and Neuroscience, University of Melbourne, Victoria 3010, Australia; ‡ School of Biological Sciences, Monash University, Clayton, Victoria 3800, Australia; § Sir Peter MacCallum Department of Oncology, Department of Biochemistry and Molecular Biology, University of Melbourne, Victoria 3010, Australia; ** Department of Biochemistry and Genetics, La Trobe Institute of Molecular Sciences, La Trobe University, Melbourne, Victoria 3086, Australia

**Keywords:** cell polarity, *Drosophila*, phosphoprotein, kinase, phosphatase, Hippo, Wingless, Wnt, Ras, phosphoinositol, PI3K, nutrient sensing

## Abstract

In both *Drosophila melanogaster* and mammalian systems, epithelial structure and underlying cell polarity are essential for proper tissue morphogenesis and organ growth. Cell polarity interfaces with multiple cellular processes that are regulated by the phosphorylation status of large protein networks. To gain insight into the molecular mechanisms that coordinate cell polarity with tissue growth, we screened a boutique collection of RNAi stocks targeting the kinome for their capacity to modify *Drosophila* “cell polarity” eye and wing phenotypes. Initially, we identified kinase or phosphatase genes whose depletion modified adult eye phenotypes associated with the manipulation of cell polarity complexes (via overexpression of Crb or aPKC). We next conducted a secondary screen to test whether these cell polarity modifiers altered tissue overgrowth associated with depletion of Lgl in the wing. These screens identified Hippo, Jun kinase (JNK), and Notch signaling pathways, previously linked to cell polarity regulation of tissue growth. Furthermore, novel pathways not previously connected to cell polarity regulation of tissue growth were identified, including Wingless (Wg/Wnt), Ras, and lipid/Phospho-inositol-3-kinase (PI3K) signaling pathways. Additionally, we demonstrated that the “nutrient sensing” kinases *Salt Inducible Kinase 2* and *3* (*SIK2* and *3*) are potent modifiers of cell polarity phenotypes and regulators of tissue growth. Overall, our screen has revealed novel cell polarity-interacting kinases and phosphatases that affect tissue growth, providing a platform for investigating molecular mechanisms coordinating cell polarity and tissue growth during development.

Apical–basal polarization of the epithelium is essential to maintain tissue architecture and restrict organ growth (Elsum and Humbert 2013). Epithelial cell polarity arises due to the creation of distinct membrane domains (apical, basal, and basolateral) via the coordinated activity of three major polarity complexes, which are conserved from flies to humans. Specifically, epithelial cell polarity is coordinated by: (1) the Crumbs (CRB) complex, comprised of the transmembrane protein Crb and associated proteins Stardust and Patj, localized at the subapical region; (2) the Scribble module [Scribble (Scrib), Disc-large (Dlg), and Lethal (2) giant larvae (Lgl)], localized to septate junctions in *Drosophila* (basolaterally in mammals) to promote basolateral membrane identity; and (3) the Partitioning defective (PAR) complex [atypical protein kinase C (aPKC), Bazooka (PAR3), and PAR6)], which promotes the separation of basolateral and subapical membrane domains (Tepass 2012). Dynamic and reciprocal interactions between these polarity complexes determine cellular membrane identity and epithelial organization (McCaffrey and Macara 2011).

A critical determinant of cell polarity is the activity of the PAR complex and aPKC, which has dual roles in cell polarity. aPKC directly phosphorylates (1) Crb to allow binding of the Std/Patj complex (Sotillos *et al.* 2004) and (2) Lgl to result in exclusion from the apical membrane (Betschinger *et al.* 2005; Plant *et al.* 2003). Additionally, these apical–basal cell polarity regulators also control tissue growth. *Drosophila lgl*, *scrib*, and *dlg* are termed “junctional scaffold” neoplastic tumor suppressor genes, mutations of which are associated with loss of cell polarity and characterized by imaginal disc epithelial and neural tissue overgrowth, impaired differentiation, and the formation of transplantable tumors (Hariharan and Bilder 2006). Despite detailed knowledge of the molecular interactions between the Crb, PAR, and Lgl complexes in the establishment and maintenance of cell polarity, how these mutually exclusive polarity modules interact to coordinate epithelial organization with tissue growth is less well understood. Thus, we have been investigating how these polarity regulators control tissue growth, and have discovered that Lgl’s, but not Scrib’s or Dlg’s, role in tissue growth control occurs via regulation of signaling pathways and that this function is independent of Lgl’s role in cell polarity (Doggett *et al.* 2011, 2015; Grzeschik *et al.* 2007, 2010a; Parsons *et al.* 2014a; Portela *et al.* 2015; Richardson and Portela 2017). Of particular relevance here, our previous studies have shown that loss of *lgl* and the concomitant increase in aPKC activity, or increased levels of Crb, impair the Hippo tissue growth control pathway and are associated with ectopic cell proliferation, decreased apoptosis, and subsequent tissue overgrowth in the *Drosophila* eye (Grzeschik *et al.* 2010a,b; Parsons *et al.* 2010, 2014a,b; Portela *et al.* 2015; Richardson and Portela 2017).

To gain insights into the relationship between epithelial structure and organ growth, we utilized cell polarity phenotypes in the adult *Drosophila* eye and undertook a boutique genetic screen using RNA interference (RNAi). Due to the critical role phosphorylation plays in regulating the activity of numerous cellular signaling processes and growth pathways, we screened a collection of kinase and phosphatase RNAi lines. By screening for modification of the adult eye phenotypes due to overexpression/activation of Crb or aPKC (using *GMR > crb^intra^* or *GMR > aPKC^CA^*), we identified 185/365 genes that were capable of modifying these phenotypes. To further explore the ability of these cell polarity modifier genes to regulate tissue growth, we extended our analysis to screen for modification of a tissue overgrowth phenotype associated with knockdown of *lgl* in the adult *Drosophila* wing [using *en > lgl-RNAi* (*lgli*)]. From this secondary screen of the 185 genes from the primary screen, we identified 18 genes that also modified the *en > lgli* adult *Drosophila* wing size, compared with *en* > alone. Of the 18 genes that modified cell polarity phenotypes in the adult *Drosophila* eye and wing, several modulated signaling pathways involved in tissue growth control, such as the Hippo, Wingless/Wnt, and inositol phosphate signaling pathways. We also identified stress responsive genes, such as members of the nutrient sensing or AMP-activated protein kinases genes *SIK2* and *SIK3* (Shackelford and Shaw 2009). In summary, our genetic screens identified genes and biological processes that provide entry points to investigate the molecular mechanisms that coordinate epithelial structure and tissue growth control during development.

## Materials and Methods

### Drosophila stocks

We used the *GAL4/UAS* system for tissue-specific expression of transgenes (Brand and Perrimon 1993): *glass multimer repeat* [*GMR - GAL4* (*II*)] and *engrailed* [*en - GAL4* or *en - GAL4*, *UAS - GFP* (*II*)] express *GAL4* predominantly in larval eye or wing discs, respectively [obtained from the Bloomington *Drosophila* Stock Center (BDSC)]. We constructed three cell polarity stocks: *GMR - GAL4* was recombined with *UAS - aPKC^CAAXWT^* (Sotillos *et al.* 2004) or *UAS - crb^intra-38.1.2b^* (Klebes and Knust 2000), and the *en > lgli* stock was generated by standard genetic techniques. *UAS - lgl - RNAi^51249^* was obtained from the Vienna *Drosophila* RNAi Center (VDRC) (Dietzl *et al.* 2007), and we have shown that it efficiently depletes Lgl and can be rescued by the human ortholog Hugl1 (Grzeschik *et al.* 2010a).

Two independent stocks of each genotype were used to screen for modifiers.

$$GMR>aPKC^{CA}:GMR-GAL4,UAS-aPKC^{CAAXWT}/CyO.$$

$$GMR>crb^{\mathrm{int}ra}:\mathrm{GMR}-GAL4,UAS-crb^{\mathrm{int}ra-\mathrm{38}\mathrm{.1}\mathrm{.2b}}/CyO.$$

$$en>\mathrm{lg}li:en-GAL4,UAS-GFP/CyO;UAS-\mathrm{lg}l-RNAi^{\mathrm{51249}}/TM6B.$$

*UAS - SIK3^K70M^* and null allele *SIK3^72^* were obtained from M. Montminy, Salk Institute for Biological Studies, California.
*UAS - 40D* (*UAS^40D^*) was obtained from the VDRC (60101).
*naked-lacZ* (*P*(*ry*[*+7.2*]*=PZ*)*nkd*[*04869a*]) and independent *SIK3* RNAi stock TRiP.JF03002 were obtained from the BDSC (#25111 and #28366 respectively).

### Genetic screens

**Figure 1:**
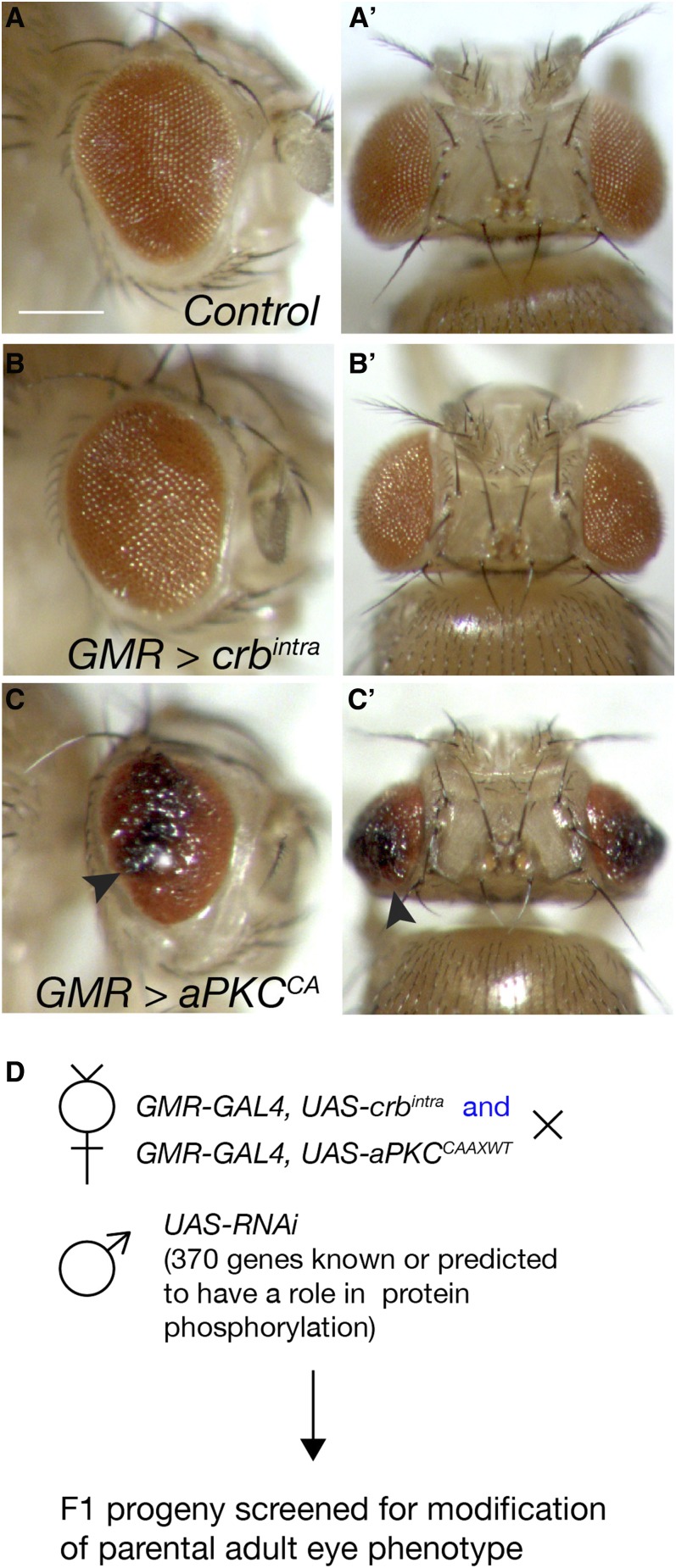
Overexpression of cell polarity components in the developing *Drosophila* eye alters adult eye structure. (A–C’) Adult female eye images, side and dorsal views, respectively. Posterior to the left in (A–C). White bar, 250 μM. (A and A’) Control (*GMR-GAL4*) adult eyes displaying highly organized, regular, lattice-like organization of ommatidia to form the adult retina. (B and B’) *GMR > crb^intra^* adult eyes are slightly larger and rougher than control eyes. (C and C’) *GMR > aPKC^CA^* adult eyes are smaller, areas of the retina show disruptions in retinal architecture and necrosis (arrowheads). (D) Genetic scheme of F1 cell polarity modifier screen. Virgin females expressing components of cell polarity complexes in the developing eye (*GMR > crb^intra^* or *GMR > aPKC^CA^*) were crossed to males carrying *UAS-RNAi* transgenes to deplete proteins with roles in protein phosphorylation. F1 progeny were scored for modification of parental adult eye phenotypes. RNAi, RNA interference; UAS, upstream activating sequence.

Genetic screens are described in Figure 1 and Figure 3. Two virgin females from *GMR > aPKC^CA^* or *GMR > crb^intra^* stocks were crossed to two males carrying *UAS - RNAi* transgenes. Two virgin females from *en > lgli* stocks were crossed to two males from *UAS - RNAi* lines that showed a modification of the *GMR > aPKC^CA^* and/or *GMR > crb^intra^* phenotype. RNAi fly stocks were obtained from the National Institutes of Genetics (NIG-Fly, Japan) or the VDRC (Supplemental Material, Table S1 in File S1). At least 30 adult F1 flies were scored for each cross, representative images are shown. All flies were raised on standard cornmeal agar food at 25° unless stated otherwise. RNAi lines that modified *GMR > aPKC^CA^* adult eyes or *en > lgli* adult wings were rescreened at 18° or room temperature, respectively, as both phenotypes were weaker at lower temperatures.

### Analysis of adult eye phenotypes

F1 progeny from crosses of *GMR > aPKC^CA^* or *GMR > crb^intra^* to *UAS - RNAi* were scored for modification of parental eye phenotypes. Adult eyes showing modification were imaged with a Scitec Infinity1 camera. Images were processed through Adobe Photoshop CS2 and Adobe Illustrator CS2.

### Analysis of wing size

F1 progeny from crosses of *en > lgli* to *UAS - RNAi* were scored for modification of parental wing phenotype. Adult wings were mounted in Canada Balsam/Xylene (Sigma) and imaged with an Olympus Stereomicroscope connected to a Scitec Infinity1 camera. Total wing area was measured with Adobe Photoshop CS2. Wing images were processed using Adobe Photoshop and Illustrator CS6.

### Statistical analysis

All statistical tests were performed separately for each data set of wing sizes. Probability values were calculated using an unpaired *t*-test with Welch’s correction to reject the null hypothesis (variation of wing size through random, independent actions of *UAS - RNAi* transgenes) in Graph Pad Prism. *P* < 0.05 was considered statistically significant.

### Gene ontology (GO) term (biological function) analysis

To functionally annotate gene lists and identify enriched GO term classes from genetic modifiers, the Princeton University web based tool, generic GO term finder, was used.

### Signaling pathway analysis

The online pathway annotation tools DAVID (Database for Annotation, Visualization and Integrated Discovery, https://david.ncifcrf.gov), PANTHER (Protein ANalysis THrough Evolutionary Relationships, http://www.pantherdb.org), and KEGG (Kyoto Encyclopedia of Genes and Genomes, http://www.genome.jp/kegg) were used to place modifier genes into functional groups.

### Immunohistochemistry

For analysis of third-instar larval wing discs, discs were dissected in phosphate-buffered saline (PBS), fixed in 4% paraformaldehyde, washed in PBS + 0.1% Triton X-100 (PBT), and blocked in PBT + 2% normal goat serum. Antibodies used were mouse anti-β−galactosidase (1:500; Rockland), rabbit anti-Lgl (1:500; Dennis Strand, Johannes Gutenberg University, Germany), and Alexa Fluor-conjugated 561, (1:500; Abcam). Confocal images were taken with an Olympus FV 1000, processed through Fiji and Adobe Photoshop CS6, and assembled in Adobe Illustrator CS6.

### Data availability

*Drosophila* stocks and antibodies are available upon request or from stock centers as listed in the *Materials and Methods*. File S1 contains four supplemental figures and seven supplemental tables.

## Results and Discussion

### Primary adult eye screen

The primary RNAi screen for modifiers of cell polarity phenotypes was conducted using the adult *Drosophila* eye, which has a regular, lattice-like structure due to the repetitive organization of groups of epithelial retinal cells (Figure 1A). The organized structure of the *Drosophila* retina makes it sensitive to defects in epithelial structure and tissue growth, and therefore an ideal system for genetic modifier screens. When compared with control (*GMR - GAL4*/+, Figure 1, A and A’), overexpression of the transmembrane–intracellular domain of *crb* (*UAS - crb^intra^*) in the posterior region of the developing larval and pupal eye (using *GMR - GAL4*) results in weak overgrowth and lens defects in the adult eye (Figure 1, B and B’) (Grzeschik and Knust 2005; Grzeschik *et al.* 2010a; Johnson *et al.* 2002; Robinson *et al.* 2010). Expression of membrane-tethered constitutively-active *aPKC* (*aPKC^CAAXWT^*, hereafter referred to as *aPKC^CA^*) via *GMR - GAL4* resulted in a small and rough adult eye with necrosis (arrowhead Figure 1, C and C’). We and others have previously demonstrated the utility of *GMR > crb^intra^* (*crb^intra^*) and *GMR > aPKC^CA^* (*aPKC^CA^*) to detect genes capable of modifying cell polarity phenotypes (Grzeschik *et al.* 2010a; Ogawa *et al.* 2009; Parsons *et al.* 2010, 2014a,b; Robinson *et al.* 2010). Thus, we conducted an F1 modifier screen to detect genes capable of altering the morphology and/or growth of the *crb^intra^* and/or *aPKC^CA^* adult eye phenotypes to identify novel factors connecting cell polarity to tissue architecture and growth.

**Figure 2:**
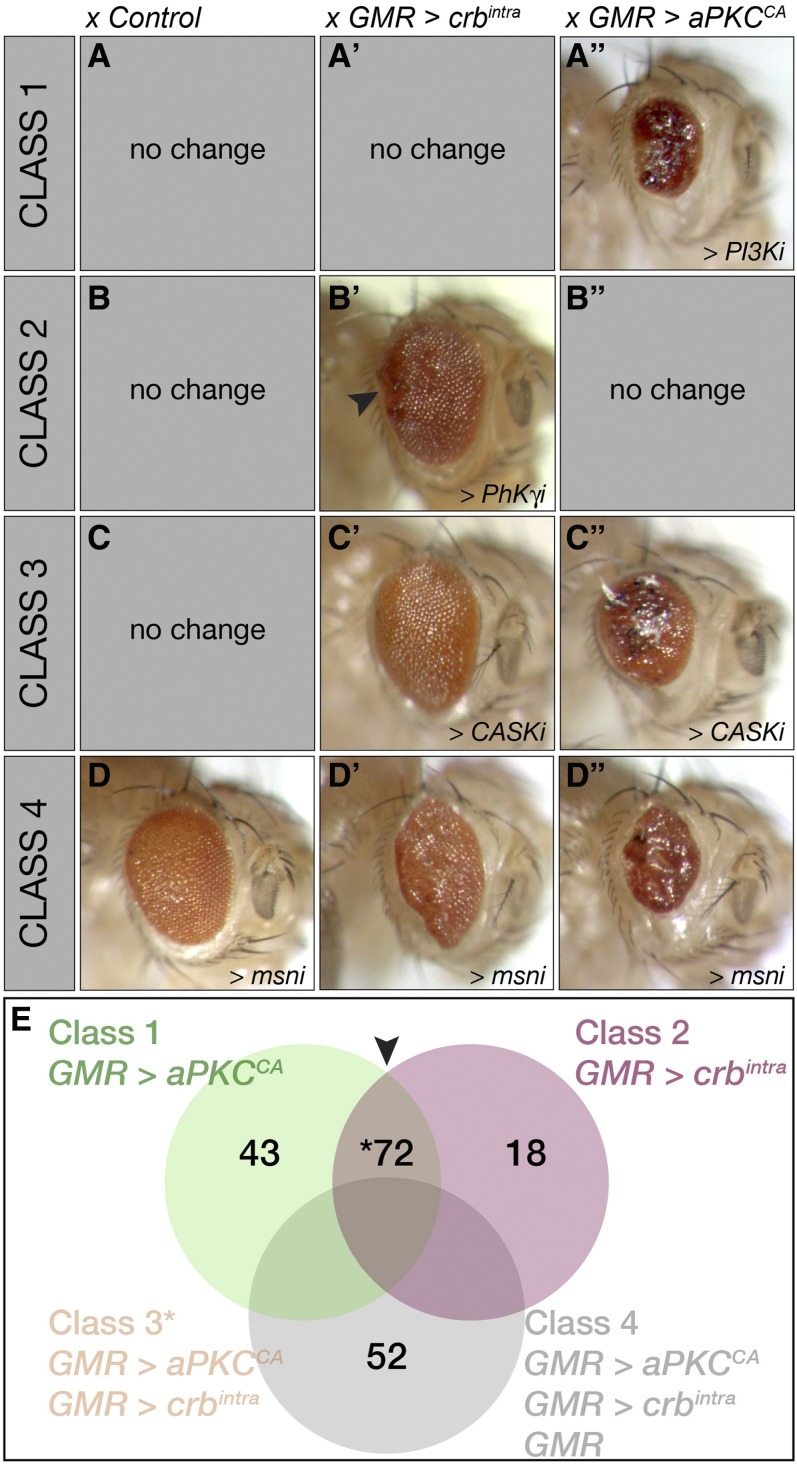
Classification of adult *Drosophila* eye cell polarity modifiers. (A–D”) Side view, adult female eyes, posterior to the left. Classification of genetic modifiers was based on genetic interaction with *GMR > crb^intra^* and/or *GMR > aPKC^CA^* and if RNAi expression alone via *GMR-GAL4* also resulted in an aberrant eye phenotype. Classes of interactors are indicated. (A–A”) Class 1: modifier genes only interacted with *GMR > aPKC^CA^*. (A) Adult eyes expressing *UAS-PI3Ki* (VDRC 38985) showed no interaction with *GMR-GAL4* or (A’) *GMR > crb^intra^* but modified (A”) *GMR > aPKC^CA^* to generate a small, glassy eye with decreased necrotic areas. (B–B”) Class 2: modifier genes only interacted with *GMR > crb^intra^*. (B) Adult eyes expressing *UAS-PhK*γ*i* (VDRC 33054) showed no interaction with *GMR-GAL4* or (B”) *GMR > aPKC^CA^* but modified (B’) *GMR > crb^intra^* to generate a larger eye with slight ruffling at the posterior edge (arrowhead). (C–C”) Class 3: modifier genes interacted with both *GMR > crb^intra^* and *GMR > aPKC^CA^*. (C) Adult eyes expressing *UAS-CASKi* (VDRC 104793 and 34184) showed a normal phenotype with *GMR – GAL4* alone but modified (C’) *GMR > crb^intra^* to generate slightly larger eyes and (C”) *GMR > aPKC^CA^* to reduce necrosis. (D–D”) Class 4: genes modified all three parental phenotypes. (D) Adult eyes expressing *UAS-msni* (NIG 1697R-1) interact with *GMR-GAL4* to generate glassy eyes. (D’) *UAS-msni* also modified *GMR > crb^intra^* to produce elongated rough adult eyes and (D”) modified *GMR > aPKC^CA^* to generate small, glassy eyes with reduced areas of necrosis. (E) Venn diagram depicting the number of genes in each Class. Green shading denotes Class 1, *GMR > aPKC^CA^*. Pink shading represents Class 2, *GMR > crb^intra^*. Brown central region (arrowhead) overlapping Class 1 and 2 represents Class 3 genes that modified both *GMR > crb^intra^* and *GMR > aPKC^CA^*. Gray shading denotes Class 4, genes that interacted with *GMR > crb^intra^* and *GMR > aPKC^CA^* and also produced an aberrant eye phenotype when expressed alone via *GMR-GAL4*. RNAi, RNA interference; UAS, upstream activating sequence; VDRC, Vienna *Drosophila* RNAi Center.

Since epithelial structure and cell polarity are integrated with cellular networks controlled by phosphorylation, we focused on genes predicted to encode kinases, phosphatases, and associated factors (Tables S1 and S2 in File S1, respectively). To identify those factors capable of genetically interacting with *crb* and/or *aPKC*, we conducted an F1 screen for modifiers of the *crb^intra^* and/or *aPKC^CA^* adult eye phenotypes using transgenic *UAS - RNAi* hairpin lines targeting 365 kinases or phosphatases (Figure 1D). From this screen, we identified 185 genes that modified the adult *Drosophila* cell polarity eye phenotypes. The 185 genes identified were grouped into four classes based on their interaction with *crb^intra^* and/or *aPKC^CA^* and/or *GMR – GAL4* alone. Class 1 genes only modified *aPKC^CA^* [*e.g.*, *Dp110* (*Pi3K92E*); Figure 2, A–A”]. Class 2 genes only modified *crb^intra^* [*e.g.*, calcium calmodulin regulated kinase *PhK*γ; Figure 2, B–B”). Class 3 genes modified both *aPKC^CA^* and *crb^intra^* but did not generate a phenotype with *GMR - GAL4* alone (*e.g.*, membrane-associated guanylate kinase *CASK*; Figure 2, C–C”). Class 4 hits not only interacted with with *crb^intra^*, *aPKC^CA^*, but their knockdown alone with *GMR - GAL4* resulted in a visible phenotype [*e.g.*, JNK pathway member *misshapen* (*msn*); Figure 2, D–D”]. Because these RNAi lines crossed to *GMR - GAL4* generate an eye phenotype, it is possible that some of these genes may be false positive Crb and aPKC modifiers; however, so as to not miss any Crb and aPKC interactors, we proceeded with the analysis of this class of interactors. Of the 185 genes identified, 43 belonged to Class 1, 18 belonged to Class 2, 72 belonged to Class 3, and 52 were in Class 4 (Figure 2E). For a full list of genes identified in the screen and a brief description of their phenotype alone and/or phenotypic modification of *crb^intra^* and/or *aPKC^CA^* see Table S3 in File S1. The high proportion of genetic interactions (51% of genes screened) suggests that phosphoprotein networks play in important role in epithelial organization and/or tissue growth control together with aPKC and/or Crb in *Drosophila* eye development. Interestingly, the CMGC family of kinases (CDK, MAPK, GS3K, and CLK), comprising protein kinases involved in the MAPK cascade and mitotic cell cycle (Table 1), is the most highly enriched, suggesting that these kinases might play important roles in aPKC and/or Crb function.

**Table 1:** Frequency analysis of GO terms Class 1–4 modifiers of *GMR > crb^intra^* and *GMR > aPKC^CA^*

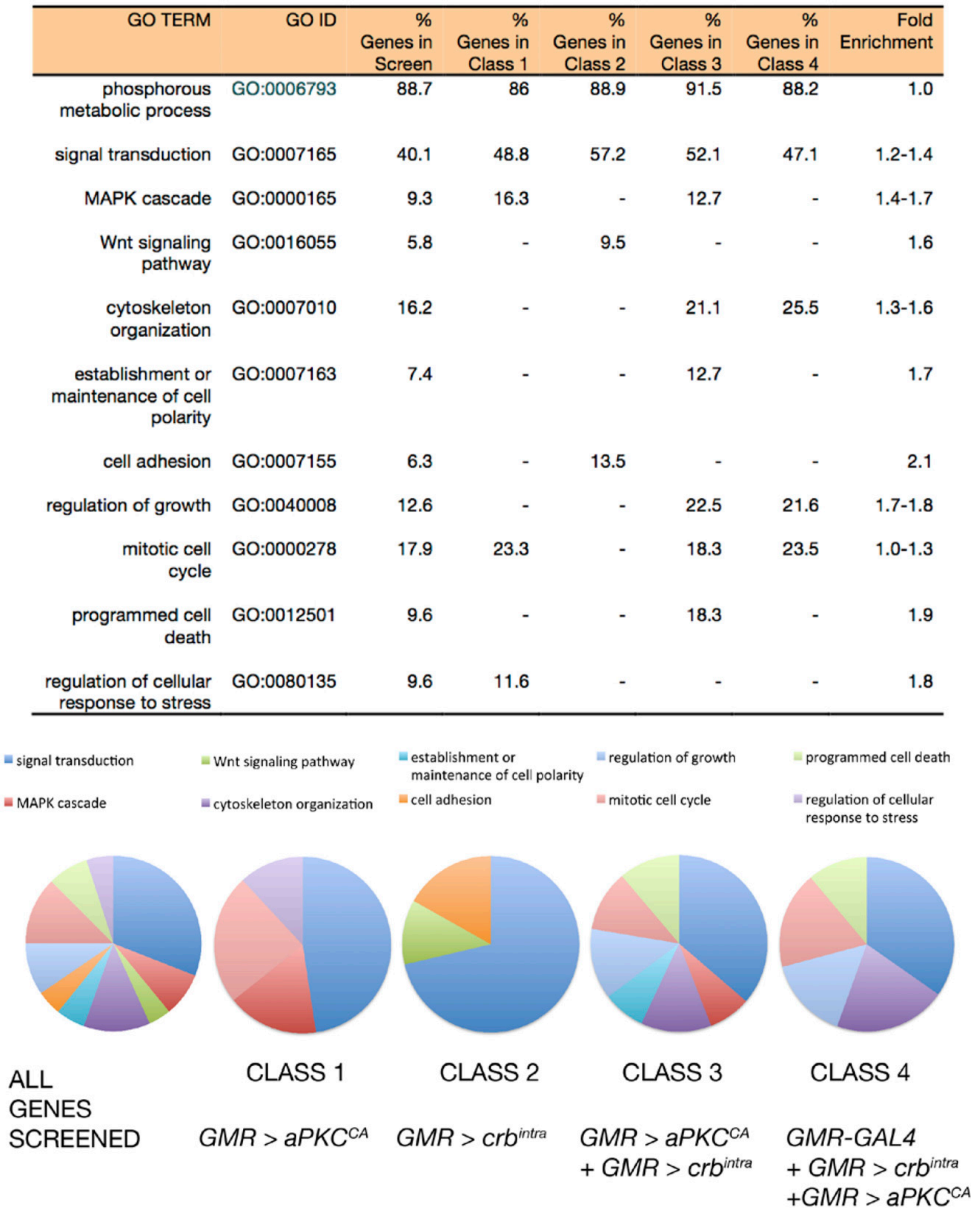

The frequency of GO terms in Classes 1–4 is shown as a percentage of genes within the individual class that associate with the GO term. Fold enrichment was calculated by dividing the percentage of genes associated in individual classes by the percentage of genes in the total number of genes screened. Fold enrichment range reflects lowest to highest enrichment from all four Classes. Similarities and differences in GO term frequency between Classes are depicted graphically in pie charts (see Legend for color coding of GO terms analyzed). Individual pie charts for Classes 1–4 lack the range of GO terms observed in the total pool of genes screened, indicating that the cell polarity phenotypes selectively interacted with modifiers. Moreover, each Class has different GO term association patterns suggesting that the cell polarity phenotypes are not equivalent and therefore may impact different signaling networks.

### GO analysis of adult Drosophila eye cell polarity modifier genes

As the starting population of genes for this boutique screen was highly enriched for GO terms, such as phosphorous metabolic process genes (323/365, 88.7%), it was not possible to detect GO term enrichment for highly represented terms from Class 1–4 gene sets. Nevertheless, examination of GO terms with lower levels of enrichment between Classes 1–4, compared with the starting pool of genes screened, revealed modest enrichment of GO terms between the starting pool and modifier genes (Table 1). Classes 1–4 all showed different distributions for the 10 GO terms analyzed (Table 1). Genes associated with the GO term “establishment or maintenance of cell polarity” were only found in Class 3 (Table 1), highlighting the sensitivity of both the *aPKC^CA^* and *crb^intra^* adult eye phenotypes to changes in cell polarity network activity. Although all classes showed association with the GO term “signal transduction,” only classes with *aPKC^CA^* modifiers showed enrichment for MAPK signaling pathway genes. Furthermore, depletion of genes associated with “regulation of cellular response to stress” was only observed in Class 1 (*aPKC^CA^*). Taken together, these genetic screens demonstrated that cell polarity network activity was sensitive to several cellular processes, including proliferation, stress, and signaling pathways; however, distinct cell polarity modules may have different sensitivities and responses to these inputs.

### Secondary adult wing screen

**Figure 3:**
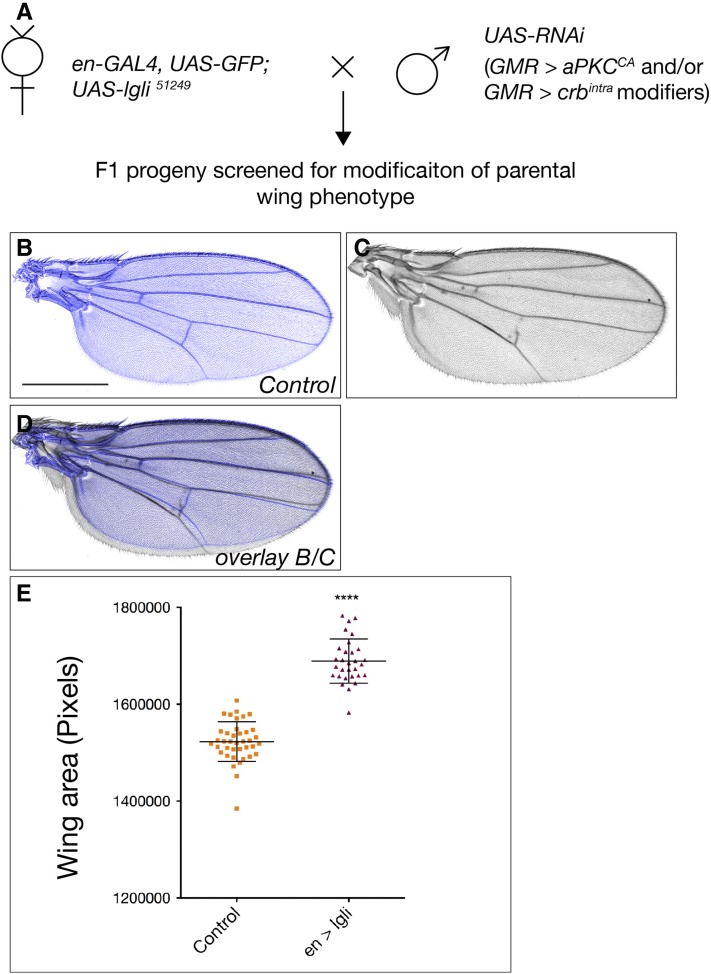
Depletion of *lgl* in the developing *Drosophila* wing increases adult wing size. (A) Genetic scheme of F1 cell polarity modifier screen. Virgin females expressing RNAi depletion of Lgl (*en > lgli*) were crossed to males carrying *UAS-RNAi* transgenes corresponding to the 185 genes identified in the primary screen. F1 progeny were scored for modification of parental adult wing size. Black bar, 500 μM. (B, C, and D) Adult female wings, anterior up, proximal to the left. (B) Control adult wing. (C) Adult wing *en > UAS-GFP*; *UAS-lgl-RNAi* (VDRC 51249) displayed increased growth. (D) Overlay of (B) and (C) highlighting *en > lgli* overgrowth (gray). (E) Quantification of total wing area in control (*en > GFP*, *RFP*) flies compared to *en > lgli*. Results represent individual wings ± SD. **** *P* < 0.0001. RNAi, RNA interference; UAS, upstream activating sequence.

Examination of gene lists corresponding to RNAi lines that modified *aPKC^CA^* and/or *crb^intra^* (Classes 1–4) revealed numerous genetic interactors that might be new genes involved in cell polarity regulation of tissue growth (Table S3 in File S1). To confirm these genetic interactions, as well as to reveal genes involved in linking cell polarity regulation to tissue growth, we conducted a secondary genetic screen where we upregulated aPKC and Crb in the developing wing by knocking down Lgl. Due to the antagonistic interaction between Lgl and aPKC, knockdown of Lgl results in increased aPKC activity (Betschinger *et al.* 2005), which in turn phosphorylates and activates the Crb complex (Fletcher *et al.* 2012; Sotillos *et al.* 2004). We used the wing epithelium, rather than the eye, since it is easier to quantify effects on tissue growth in the adult wing than in the eye, as well as to reveal genes that interact with deregulated Lgl/aPKC and Crb in another epithelial tissue. To knockdown Lgl, we used the *engrailed* (*en*)-*GAL4* driven expression of a *UAS-lgl-RNAi* line, which is expressed in the posterior compartment of the developing wing from embryogenesis (Figure 3A). In this wing model, we quantified altered tissue growth by measuring adult wing size. Depletion of Lgl in the posterior half of the developing wing disc [*en - GAL4* driven *UAS - lgl-RNAi* (*en > lgli*)] (Figure S1 in File S1) resulted in a 10% increase in total adult wing area (Figure 3, B and C, overlayed in Figure 3D, and quantified in Figure 3E). Thus, we conducted a secondary screen of the 185 cell polarity modifier hits for those able to modify the wing overgrowth due to Lgl depletion.

**Figure 4:**
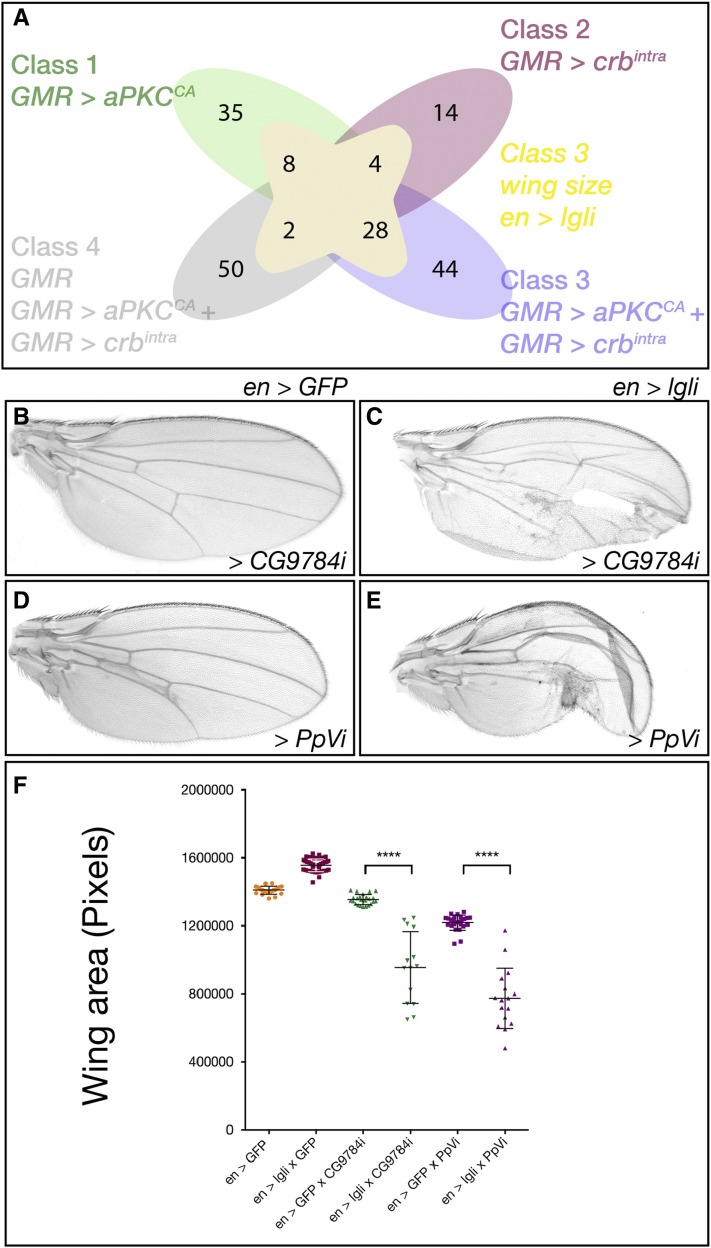
Modifier genes of *en >lgli* adult wing size. (A) Venn diagram depicting the number of modifier genes in Classes 1–4 from the adult eye screen (total 185) and overlap between modifier genes identified in *en > lgli* wing size Class 3 (total 42). Central yellow shading denotes *en > lgli* wing size Class 3 modifiers. Green shading denotes eye Class 1, *GMR > aPKC^CA^*. Pink shading denotes eye Class 2, *GMR > crb^intra^*. Purple denotes eye Class 3 genes that modified: *GMR > crb^intra^* and *GMR > aPKC^CA^*. Gray shading denotes eye Class 4, genes that interacted with *GMR > crb^intra^* and *GMR > aPKC^CA^* but also show a phenotype when expressed alone via *GMR-GAL4*. The overlap with *en >lgli* (yellow) indicates the number of genes from each eye Class that also modified *en >lgli* wing size Class 3. (B–E) Adult female wings, anterior up, proximal to the left. (B) *en > lgli* specific modifier gene: *en > GFP*; *CG9784i* (VDRC 30098) wings show no change in wing size. (C) *en > lgli/CG9784i* have decreased wing growth and holes. (D) *en > lgli* modifier gene: *en > GFP*; *PpVi* (VDRC 101997) wings are slightly smaller than the control (<5%) yet significantly impacted (E) *en >lgli* adult wing growth. (F) Quantification of total wing area (A–D) in control animals compared to *en > GFP* and *en > lgli*. Results represent individual wings ± SD. **** *P* < 0.0001. VDRC, Vienna *Drosophila* RNAi Center.

Of the 185 positive hits identified in the adult eye screen, 93 also interacted with *en > lgli* or *en-GAL4* alone (Figure 4A). *en > lgli* interactors were broadly classified into three groups (Table S4 in File S1). Class 1 comprised 41 genes that resulted in pupal lethality with *en > lgli* and/or *en – GAL4* alone, which precluded the specific effect of the gene knockdown on the wing growth being analyzed. Class 2 included 10 genes that produced adults following coknockdown of *lgl*, but the crumpled wing phenotype precluded measurement. The most informative class, Class 3, comprised 42 genes, where either codepletion with *lgl* and/or depletion alone altered adult wing size. Essentially all Class 3 genes, except *Tao-1* and *SIK3*, suppressed adult wing growth associated with *lgl* depletion.

After we had completed our screens, we were alerted to a recent report suggesting that ∼25% of the VDRC KK RNAi collection can generate false positive enhancement of impaired Hippo pathway signaling, due to ectopic expression of the *tiptop* (*tio*) transcription factor gene from the *40D* insertion site (Vissers *et al.* 2016). To determine if aberrant *tio* expression might be influencing our screen results, we tested the polarity phenotypes with the *tio* tester stock (*40D^UAS^*). We observed modification of the *GMR > crb^intra^*, *en - GAL4*, and *en > lgli*, but not *GMR - GAL4* or *GMR > aPKC^CA^*, with the *tio* tester stock (*40D^UAS^*) (Figure S2 in File S1). We note that in some instances where two or more RNAi lines for a given gene were tested for modification of *crb^intra^* and *aPKC^CA^*, the genetic interaction produced opposite results [*e.g.*, *wunen* (*wun*), Table S3 in File S1] this may be due to a false positive interaction of the KK line with *crb^intra^*, off-target effects, or the ability of different RNAis to efficiently suppress target genes. As KK lines interacting with both *crb^intra^* and *lgli* may represent false positives, we intersected *crb^intra^* and *lgli* modifiers, which revealed four interactors: *CG1830* (*PhK*γ), *CG10417*, *CG8866*, and *CG32484* (*Sk2*) (Figure 4A and Table S5 in File S1). Three of these interactors, *CG10417*, *CG8866*, and *CG32484* (*Sk2*), were identified with KK lines and may represent false positives that need further verification by testing with independent RNAi lines.

Systematic analysis of wing sizes of *en > lgli* compared with *en – GAL4* interactors in Class 3 revealed four subclasses: Subclass 3.1 interactors only modified *en > lgli*; Subclass 3.2 modifiers affected *en – GAL4* and *en > lgli* wing size equivalently; Subclass 3.3 *en > lgli* wings were smaller than *en – GAL4* modified wings; and Subclass 3.4 *en > lgli* wing sizes were larger than *en – GAL4* wings. Since genes in Subclass 3.2 modified *en – GAL4* and *en > lgli* wing size equivalently, these genes were ruled out as being specific *en > lgli* interactors, leaving 18 genes in the remaining classes as Lgl modifiers. Additionally, as both *en - GAL4* and *en > lgli* crossed to *40D^UAS^* wings displayed an ∼10% decrease in wing size (Figure S2 in File S1), 15 Subclass 3.2 wing modifiers (where equivalent reduction in *en > lgli* and *en* wing growth was observed) may also be false positives (indicated with *, Table S6 in File S1). In summary, the *en > lgli* screen identified 18 kinases and phosphatases where knockdown of the modifier gene only showed modification of wing growth with *lgli* but not with *en*, or modified *en > lgli* wing size more than *en* alone (Table S6 in File S1). These genes include *CG9784* (*IPP*, lipid phosphatase) and *PpV* (predicted Wnt pathway regulator; Swarup *et al.* 2015), which dramatically modified Lgl-depleted wings, but had little effect or ∼10% reduced growth following knockdown alone (Figure 4, B–E, respectively, quantified in Figure 4F). Thus, although screening the VDRC KK RNAi collection can generate false positive genetic interactions and some modifier genes require further validation (see Table S6 in File S1), we have identified 18 kinase/phosphatase genes that might coordinate cell polarity cues and tissue growth signals during organ development.

Overlap between modifier genes that specifically affected adult eye cell polarity phenotypes (Classes 1–3) and the *en > lgli* wing size (Classes 3.1, 3.3, and 3.4) (Figure S3 and Tables S6 and S7 in File S1) revealed that 15 of the Lgl wing size modifier genes interacted with both aPKC and Crb, suggesting that these genes were general cell polarity tissue growth regulators. Three genes, *Btk29A*, *Ror*, and *PpY-55A*, did not interact with *Crb*, suggesting that these genes might be specific for the *Lgl–aPKC* axis of the tissue growth regulatory pathway. For simplicity, we will henceforth refer to these 18 genes as cell polarity–tissue growth interactors.

### Cell polarity–tissue growth-interacting genes are associated with many signaling pathways

**Figure 5:**
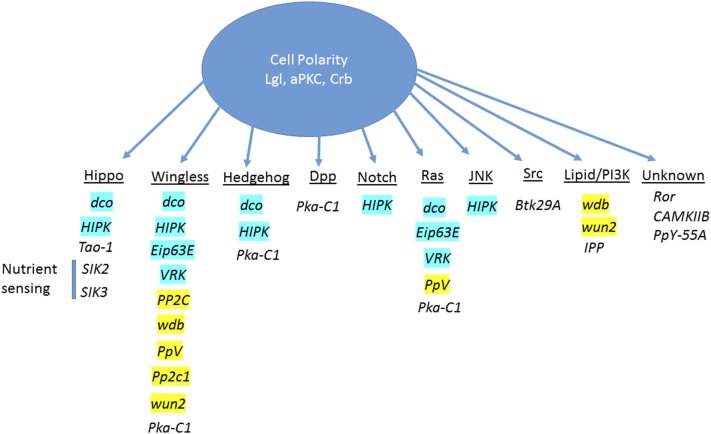
Summary of cell polarity tissue growth interactors and the signaling pathways they are associated with. Genes from the wing size Class 3 that modified *en > lgli*, greater than *en-GAL4* alone (Classes 3.1, 3.3, and 3.4), are listed under the signaling pathways that they are associated with. Genes highlighted in blue or yellow were identified as kinases or phosphatase regulators of Wnt signaling respectively (Swarup *et al.* 2015). JNK, Jun kinase; PI3K, phospho-inositol-3-kinase.

Analysis of the 18 cell polarity–tissue growth interactors (Classes 3.1, 3.3, and 3.4) for their links to signaling pathways (Figure 5 and Table S7 in File S1), revealed that many were associated with signaling pathways that Lgl, aPKC, or Crb have previously been shown to regulate in tissue growth control: the Hippo, Notch, and JNK pathways (Grzeschik *et al.* 2010a; Parsons *et al.* 2014a,b; Portela *et al.* 2015; Sun and Irvine 2011; Zhu *et al.* 2010). Notably, other Lgl-interacting genes were associated with signaling pathways not previously linked to the negative regulation of tissue growth by Lgl: Wg, Decapentaplegic (Dpp), Hedgehog (Hh), Src, Ras, and lipid signaling/PI3K (Figure 5 and Table S7 in File S1). However, three Lgl-interacting genes, the protein kinase genes *Ror* and *CAMKIIB* and the phosphatase gene *PpY-55A*, have been poorly studied and have not yet been associated with known signaling pathways.

Several of the cell polarity–tissue growth interactors affected the Hippo signaling pathway; depletion of *Tao-1* (Class 3.4) (Boggiano *et al.* 2011; Poon *et al.* 2011), *Hipk* (Class 3.3) (Chen and Verheyen 2012), *dco* (Class 3.1) (Milton *et al.* 2010; Sopko *et al.* 2009), *SIK2* (Class 3.3), and *SIK3* (Class 3.4) (Wehr *et al.* 2013) modified the Lgl, aPKC, and Crb cell polarity phenotypes. Interestingly, the founding members of the Hippo pathway, *hpo* and *wts* (Class 3.1) (Harvey and Tapon 2007), also interacted with *en > lgli*, resulting in pupal lethality, although at lower temperatures *hpo-RNAi* produced adults with wing size defects (Table S4 in File S1). Since Lgl, aPKC, and Crb are known to regulate the Hippo pathway (Chen *et al.* 2010; Grzeschik *et al.* 2010a; Ling *et al.* 2010; Parsons *et al.* 2010; Robinson *et al.* 2010), the identification of Hippo pathway regulators in our *en > lgli* screen demonstrates that this phenotype is sensitive to modifier genes that regulate tissue growth.

We have previously demonstrated that Notch signaling is impaired in *lgl* mutant eye epithelial tissue and contributes to the tissue growth effects (Parsons *et al.* 2014a; Portela *et al.* 2015), and Crb has also been shown to regulate Notch signaling in the eye (Richardson and Pichaud 2010). *HIPK*, identified as an *lgl*, *aPKC*, and *crb* interactor, in addition to its regulation of Hippo pathway signaling, also acts a positive regulator of Notch signaling in eye development (Lee *et al.* 2009). HIPK also regulates JNK signaling in the *Drosophila* wing epithelium (Huang *et al.* 2011), and in this tissue Lgl depletion-mediated cell polarity and tissue growth effects are JNK-dependent (Sun and Irvine 2011; Zhu *et al.* 2010). Thus, HIPK’s role in regulating JNK signaling in the wing tissue and in Notch signaling in the eye tissue might also underlie its genetic interactions with *lgl*, *aPKC*, and *crb*. However, although core members of the JNK pathway, Bsk, Tak1, Takl2, and Misshapen (Msn), were identified as modifiers of the *GMR > aPKC* or *GMR > aPKC* and *GMR > crb* eye phenotypes (Table S3 in File S1), knockdown of Tak1 or Msn were lethal with *en > lgli* and *en>*, precluding analysis of their specific interaction with *lgl*, while Bsk or Takl2 knockdown did not noticeably modify the *en > lgli* phenotype (Table S4 in File S1).

Our analysis of the Lgl-interacting genes revealed several novel signaling pathways (Dpp, Wg, Hh, Src, Ras, and lipid-PI3K) not previously implicated in the negative regulation of tissue growth by Lgl, aPKC, or Crb cell polarity regulators, which we will detail below.

Lgl regulates Dpp [Bone Morphogenetic Protein (BMP)] signaling in the wing epithelium by promoting the secretion of the Dpp morphogen (Arquier *et al.* 2001). Pka-C1, which genetically interacts with *lgl*, *aPKC*, and *crb* phenotypes, negatively regulates Dpp signaling (Li *et al.* 1995), so may genetically interact with Lgl, by affecting Dpp signaling. However, since Dpp positively regulates wing tissue growth (Brumby and Richardson 2005) and Lgl depletion would be expected to decrease Dpp signaling, this is unlikely to directly account for the wing size increase. Furthermore, knockdown of Pka-C1 should lead to increased Dpp signaling, as well as Hh and Wg signaling in the wing epithelium (Li *et al.* 1995), and therefore it is difficult to understand how Pka-C1 leads to a reduction in *en > lgli* wing growth. Interestingly, Pka-C1 promotes Ras-induced stem cell proliferation in the malpighian tubules (Zeng *et al.* 2010), and therefore reduced Pka-C1 might also reduce Ras signaling in the wing leading to the reduced tissue growth of *en > lgli* wings; however, further investigation is required to investigate this possibility. Interestingly, there is cross talk between the Dpp and Hippo pathways that might also impact on this interaction (Oh and Irvine 2011; Rogulja *et al.* 2008).

The Wg (Wnt) signaling pathway was associated with many of the Lgl interactors. The Wg/Wnt pathway regulates many biological processes, including proliferation and differentiation, to coordinate organ growth and planar cell polarity (Clevers and Nusse 2012). Interestingly, as measured by increased expression of the *naked* (*nkd*)*-lacZ* Wg signaling reporter (Tyler and Baker 2007; Zeng *et al.* 2000), we found that Wg signaling was upregulated in *lgl^27S3^* mutant clones relative to the surrounding wild-type cells in larval eye discs (Figure S4 in File S1). Consistent with Lgl-modulating Wg signaling, two cell polarity tissue growth interactor genes are implicated in Wg signaling [*discs overgrown* (*dco*) (Class 3.1) (Klein *et al.* 2006), and *cAMP-dependent protein kinase 1* (*Pka-C1*) (Class 3.3) (Li *et al.* 1995)]. Furthermore, comparison between the cell polarity tissue growth interactors, and kinase and phosphatase genes recently predicted to regulate Wg signaling in *Drosophila* (Swarup *et al.* 2015), revealed *dco* and three other kinase genes [*VRK* (Class 3.3), *Hipk* (Class 3.3), and *Eip63E* (Class 3.1)], as well as five phosphatases genes [**wun2**, *PP2c1*, *PpV*, *wdb* (all Class 3.3), and *PP2C* (Class 3.1)] (Figure 5 and Tables S6 and S7 in File S1). Therefore, these genes might regulate Wg signaling to modify the Lgl, aPKC, or Crb phenotypes. Consistent with the association of these interactors with the Wg pathway, previous studies have revealed genetic interactions between the Wg signaling pathway and Lgl/aPKC in *Drosophila* embryo epithelial morphogenesis (Dollar *et al.* 2005; Kaplan *et al.* 2009, 2011; Kaplan and Tolwinski 2010) and in *Xenopus* (Choi and Sokol 2009). Thus, cell polarity regulation of Wg signaling might be important in coordinating epithelial structure and organ growth. However, it should also be noted that the Wg signaling pathway can cross talk to the Hippo pathway in tissue growth control during wing development (Zecca and Struhl 2010), and therefore the effect of these cell polarity tissue growth interactors on the Wg pathway might indirectly affect the Hippo pathway to modulate the Lgl, aPKC, or Crb phenotypes.

Other signaling pathways associated with the cell polarity–tissue growth interactor genes, were Hh (*dco*, *HIPK*, and *Pka-C1*), Src (*Btk29A*), Ras (*dco*, *PpV*, *Eip63E*, *VRK*, and *Pka-C1*), lipid-PI3K [*wdb* and *CG9784* (*IPP*)], and *wun2* (Figure 5 and Tables S6 and S7 in File S1). With the cell polarity tissue growth interactors that are associated with Hh signaling, *dco* (Class 3.1; Shi *et al.* 2014), *HIPK* (Class 3.3; Swarup and Verheyen 2011), and *Pka-C1* (Class 3.3; Kiger and O’Shea 2001), two of these genes are also regulators of the Hippo pathway, and indeed Hh signaling has been linked to Hippo pathway regulation (Kagey *et al.* 2012). Thus, the link between these cell polarity tissue growth interactors and the Hh pathway may ultimately affect the Hippo pathway in the modulation of Lgl, aPKC, or Crb phenotypes. Likewise, the link between the cell polarity tissue growth interactors and the Src and Ras pathways might be also related to Hippo signaling (Enomoto and Igaki 2013; Reddy and Irvine 2013), as detailed below.

Btk29A (Class 3.1) is regulated by Src signaling in tissue growth (Read *et al.* 2004), and interestingly Src signaling has previously been shown to interact with Lgl mutant cell extrusion and invasion phenotypes in the wing epithelium (Ma *et al.* 2013). Recent studies have revealed that overexpression of Src regulates tissue growth via JNK-dependent repression of the Hippo pathway (Enomoto and Igaki 2013), and thus the suppression of the *en > lgli* wing overgrowth by *Btk29A* might be due to restored Hippo pathway signaling.

Cell polarity tissue growth interactors were also associated with the Ras pathway: *dco* (Class 3.1), *PpV* (Class 3.3), *Eip63E* (Class 3.1 (Friedman *et al.* 2011), *VRK* (Class 3.3) (Ashton-Beaucage *et al.* 2014), and *Pka-C1* (Class 3.3) (Zeng *et al.* 2010). Since elevated Ras signaling is a driver of tissue growth through promoting cell proliferation and inhibiting apoptosis (Brumby and Richardson 2005), but has been also linked to Hippo pathway impairment (Reddy and Irvine 2013), these interacting genes might indirectly affect Hippo signaling to modulate the Lgl, aPKC, and Crb phenotypes by regulating Ras signaling.

Cell polarity tissue growth interactors were also associated with lipid/PI3K signaling (Tables S6 and S7 in File S1): *CG9784* (*IPP*, encoding a Inositol phosphate phosphatase, which is involved in membrane trafficking and lipid signaling, Class 3.1) (De Craene *et al.* 2017; Erneux *et al.* 2016; Liu and Bankaitis 2010; Morrison *et al.* 2000), *wdb* (encoding PP2A-B’ subunit, which modulates PI3K-Akt signaling, Class 3.3) (Vereshchagina *et al.* 2008), and *wun2* (encoding a lipid phosphatase involved in glycerolipid metabolism, which is required for septate junction formation in the larval tracheal system, Class 3.3) (Ile *et al.* 2012). Of relevance, deregulation of phospholipid metabolism is linked to cell polarity in both mammalian cells and *Drosophila* (Claret *et al.* 2014; Shewan *et al.* 2011). Furthermore, Lgl has been recently shown to bind to PIP2 and PI4P phospholipids, which targets Lgl in *Drosophila* and mammalian cells to the plasma membrane (Dong *et al.* 2015). Thus, our data supports the growing body of research that links plasma membrane lipid domains and cell polarity. However, PI3K phosphorylates the phospho-inositol PIP2 to generate PIP3, which signals via Akt to regulate mTor (mechanistic target of rapomycin) activity in tissue growth control (Yu and Cui 2016), and mTor signaling has been recently shown to modulate target gene accessibility of the Hippo pathway effector Yki (Parker and Struhl 2015). Thus, these lipid/PI3K signaling regulators might also indirectly affect the Hippo pathway to mediate their interactions with the Lgl, aPKC, or Crb phenotypes.

Altogether, our analyses reveal several known and novel signaling pathways linking cell polarity modules to multiple regulatory networks controlling tissue growth. Many of these signaling pathways are also linked to the regulation of the Hippo pathway, therefore their interaction with Lgl, aPKC, and/or Crb may reflect their modulation of Hippo signaling. However, the precise mode by which these cell polarity–tissue growth-interacting genes modulate these signaling pathways, and in turn how they might be regulated by the polarity regulators, requires further investigation.

### Cell polarity complexes and Salt Inducible Kinase 3 interact to restrict tissue growth

**Figure 6:**
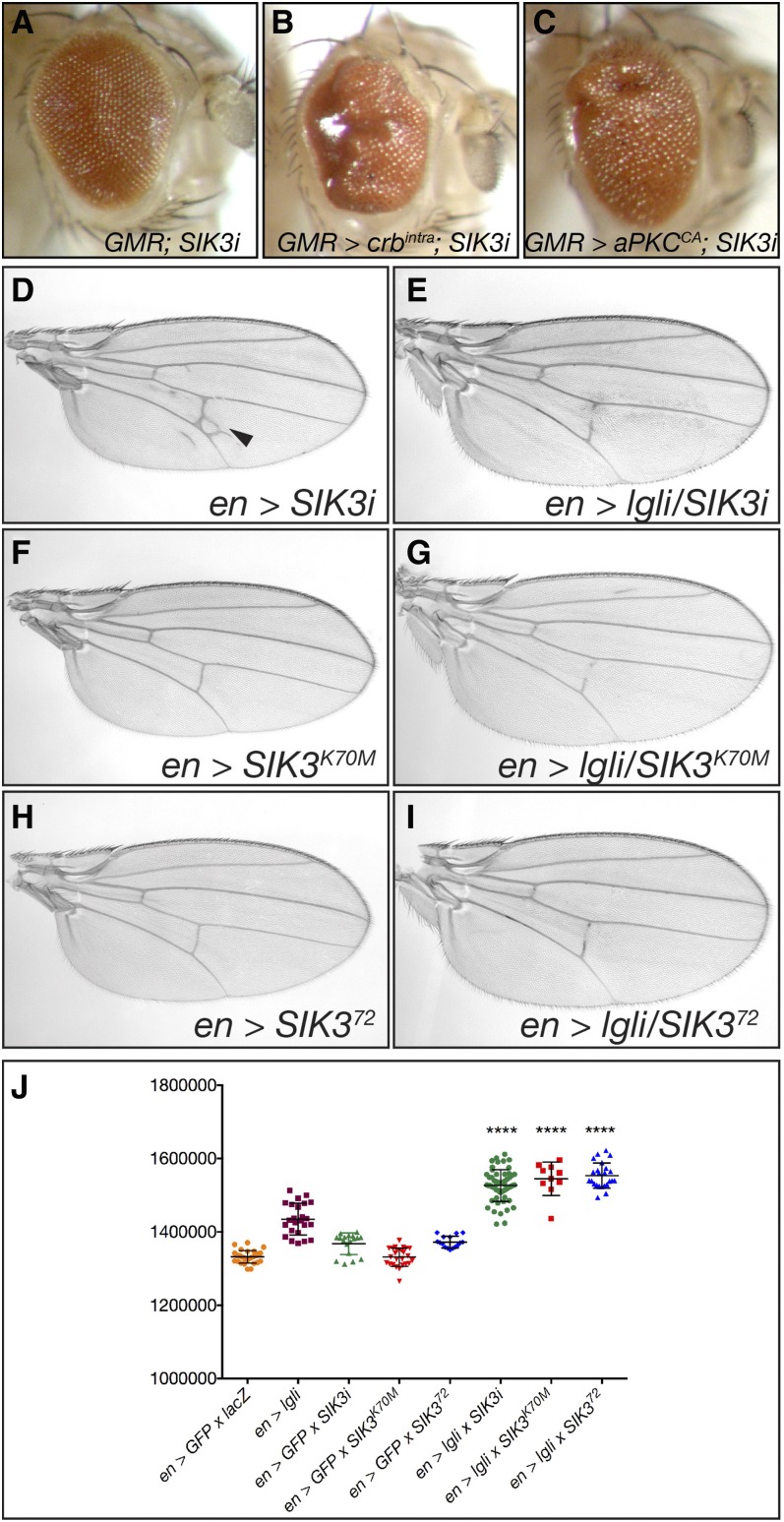
*SIK3* genetically interacts with cell polarity complexes to negatively regulate organ size. (A–C) Adult female eyes, posterior is to the left. (D–I) Adult female wings, anterior up, proximal to the left. (A) Expression of *UAS-SIK3-RNAi* (independent RNAi TRiP.JF03002) with *GMR-GAL4* in the developing eye has no effect on adult eye morphology. (B) Expression of *UAS-SIK3 RNAi* in conjunction with *GMR > crb^intra^* or (C) *GMR > aPKC^CA^* at 18° increases adult eye size. (D) Reduction of *SIK3* activity by RNAi depletion *en > GFP*; *UAS-SIK3-RNAi*, (F) overexpression of kinase dead transgene *en > GFP*; *UAS-SIK3^K70M^*, or (H) null allele *en > GFP/ SIK3^72^* has no effect on adult wing size (< 1%). (E) In conjunction with reduced *lgl* activity, *en > lgli* decreased *SIK3* activity by RNAi depletion with *UAS-SIK3-RNAi*. (G) Overexpression of kinase dead transgene (*UAS-SIK3^K70M^*) or (I) null allele *SIK3^72^* significantly increased wing size. (J) Quantification of total wing area (D–I). In D, the arrowhead indicates ectopic cross veins. Results represent individual wings ± SD. **** *P* < 0.0001. RNAi, RNA interference

Of particular interest, our cell polarity gene screens identified two members of the AMP-related kinase family, *Salt Inducible Kinase 3* (*SIK3*) (Class 3.4) and *SIK2* (Class 3.3), which have important roles in nutrient-dependent signaling (Choi *et al.* 2011, 2015; Wang *et al.* 2011), but had not previously been connected to cell polarity regulation. *SIK3* was identified in our screen as a negative regulator of tissue growth affecting aPKC, Crb, and Lgl phenotypes (Tables S4, S6, and S7 in File S1). Although depletion of *SIK3* alone did not noticeably affect adult eye morphology or size (Figure 6A), *SIK3* knockdown caused glassiness and bulging of retinal tissue in adult eyes in the *crb^intra^* or *aPKC^CA^* background (Figure 6, B and C respectively, compared with Figure 1, C and D). Wing size was unaltered following depletion of *SIK3* alone in the posterior wing compartment via RNAi (*en – GAL4* driven *SIK3i*), expression of a kinase dead transgene (*SIK3^K70M^*), or reduced *SIK3* gene dosage using *SIK3* heterozygotes (null allele *SIK3^72^*/+). However, reduced SIK3 activity, in combination with *lgl* depletion, resulted in a significant increase in wing size (compare Figure 6, D and E, Figure 6, F and G, and Figure 6, H and I; quantified in Figure J). Thus, *SIK3* modulates the activity of the cell polarity complexes and growth pathways to restrict tissue growth.

Intriguingly, our data support a requirement for *SIK2* activity in promoting normal and *lgli*-dependent wing growth (Tables S4, S6, and S7 in File S1), while reduced *SIK3* activity increased wing growth in the Lgl loss-of-function background. In *Drosophila*, Salt Inducible kinases have been implicated in tissue growth via Hippo pathway signaling (Wehr *et al.* 2013). SIK2 and SIK3 phosphorylate and inactivate Salvador (SAV), a core component of the Hippo kinase complex, leading to activation of the Yki transcriptional program and increased tissue growth (Wehr *et al.* 2013). Furthermore, codepletion of *SIK2* and *SIK3* reduced tumor growth in a *Drosophila* tumor model (activated *Src + Ras^V12^*) (Hirabayashi and Cagan 2015). Given the observation that *SIK2* and *SIK3* differentially modify Lgl-dependent tissue growth, but are both required for Hippo pathway inactivation and *Src + Ras^V12^*-driven tumor growth, future studies are required to determine how the SIKs interact with Lgl to control wing growth.

### Conclusions

Genetic screens in *Drosophila* remain a powerful tool for identifying and unraveling gene function in specific signaling pathways and cellular processes. We undertook a boutique genetic screen, specifically using RNAi lines targeting kinases and phosphatases, to identify novel signaling pathways involved in the regulation of epithelial structure with tissue growth. Analysis of the hits that genetically interacted with *lgl*, *aPKC*, and/or *crb* cell polarity genes revealed that they were associated with Hippo, Notch, JNK, Dpp, Hh, Wg, Ras, lipid/PI3K, and unknown signaling pathways. Future studies determining the molecular relationships between cell polarity proteins and the modifiers identified will be required to determine whether these interactions are direct. Recent advances in proteomics through the generation of the *Drosophila* and human Protein Interaction Map, and studies coupling genetic manipulation to the analysis of kinase–phosphatase networks, will considerably advance our capacity to define the *in vivo* function of these genes (Guruharsha *et al.* 2011; Huttlin *et al.* 2015; Sopko *et al.* 2014). Of relevance to the novel pathways revealed in this study, further exploration of the roles of *SIK2* and *SIK3* in *Drosophila* development, and mammalian cancer models, is required to unravel the intricacies between cell polarity protein complexes and nutrient sensing kinases in normal development and cancer.

## Supplementary Material

Supplemental material is available online at www.g3journal.org/lookup/suppl/doi:10.1534/g3.117.043513/-/DC1.

## References

[bib1] Arquier N. Perrin L. Manfruelli P. Semeriva M., 2001 The *Drosophila* tumor suppressor gene *lethal(2)giant larvae* is required for the emission of the Decapentaplegic signal. Development 128: 2209–2220. 11493541 10.1242/dev.128.12.2209

[bib2] Ashton-Beaucage D. Udell C. M. Gendron P. Sahmi M. Lefrancois M., 2014 A functional screen reveals an extensive layer of transcriptional and splicing control underlying RAS/MAPK signaling in Drosophila. PLoS Biol. 12: e1001809. 24643257 10.1371/journal.pbio.1001809 PMC3958334

[bib3] Betschinger J. Eisenhaber F. Knoblich J. A., 2005 Phosphorylation-induced autoinhibition regulates the cytoskeletal protein Lethal (2) giant larvae. Curr. Biol. 15: 276–282. 15694314 10.1016/j.cub.2005.01.012

[bib4] Boggiano J. C. Vanderzalm P. J. Fehon R. G., 2011 Tao-1 phosphorylates Hippo/MST kinases to regulate the Hippo-Salvador-Warts tumor suppressor pathway. Dev. Cell 21: 888–895. 22075147 10.1016/j.devcel.2011.08.028 PMC3217187

[bib5] Brand A. H. Perrimon N., 1993 Targeted gene expression as a means of altering cell fates and generating dominant phenotypes. Development 118: 401–415. 8223268 10.1242/dev.118.2.401

[bib6] Brumby A. M. Richardson H. E., 2005 Using *Drosophila melanogaster* to map human cancer pathways. Nat. Rev. Cancer 5: 626–639. 16034367 10.1038/nrc1671

[bib7] Chen C. L. Gajewski K. M. Hamaratoglu F. Bossuyt W. Sansores-Garcia L., 2010 The apical-basal cell polarity determinant crumbs regulates Hippo signaling in *Drosophila*. Proc. Natl. Acad. Sci. USA 107: 15810–15815. 20798049 10.1073/pnas.1004060107 PMC2936591

[bib8] Chen J. Verheyen E. M., 2012 Homeodomain-interacting protein kinase regulates Yorkie activity to promote tissue growth. Curr. Biol. 22: 1582–1586. 22840522 10.1016/j.cub.2012.06.074

[bib9] Choi S. Kim W. Chung J., 2011 *Drosophila* salt-inducible kinase (SIK) regulates starvation resistance through cAMP-response element-binding protein (CREB)-regulated transcription coactivator (CRTC). J. Biol. Chem. 286: 2658–2664. 21127058 10.1074/jbc.C110.119222 PMC3024761

[bib10] Choi S. Lim D. S. Chung J., 2015 Feeding and fasting signals converge on the LKB1–SIK3 pathway to regulate lipid metabolism in *Drosophila*. PLoS Genet. 11: e1005263. 25996931 10.1371/journal.pgen.1005263 PMC4440640

[bib11] Choi S.-C. Sokol S. Y., 2009 The involvement of lethal giant larvae and Wnt signaling in bottle cell formation in *Xenopus* embryos. Dev. Biol. 336: 68–75. 19782678 10.1016/j.ydbio.2009.09.033 PMC2801549

[bib12] Claret S. Jouette J. Benoit B. Legent K. Guichet A., 2014 PI(4,5)P2 produced by the PI4P5K SKTL controls apical size by tethering PAR-3 in *Drosophila* epithelial cells. Curr. Biol. 24: 1071–1079. 24768049 10.1016/j.cub.2014.03.056

[bib13] Clevers H. Nusse R., 2012 Wnt/β-catenin signaling and disease. Cell 149: 1192–1205. 22682243 10.1016/j.cell.2012.05.012

[bib14] De Craene J. O. Bertazzi D. L. Bar S. Friant S., 2017 Phosphoinositides, major actors in membrane trafficking and lipid signaling pathways. Int. J. Mol. Sci. 18: E634. 28294977 10.3390/ijms18030634 PMC5372647

[bib15] Dietzl G. Chen D. Schnorrer F. Su K. C. Barinova Y., 2007 A genome-wide transgenic RNAi library for conditional gene inactivation in *Drosophila*. Nature 448: 151–156. 17625558 10.1038/nature05954

[bib16] Doggett K. Grusche F. A. Richardson H. E. Brumby A. M., 2011 Loss of the *Drosophila* cell polarity regulator Scribbled promotes epithelial tissue overgrowth and cooperation with oncogenic Ras-Raf through impaired Hippo pathway signaling. BMC Dev. Biol. 11: 57. 21955824 10.1186/1471-213X-11-57 PMC3206446

[bib17] Doggett K. Turkel N. Willoughby L. F. Ellul J. Murray M. J., 2015 BTB-zinc finger oncogenes are required for Ras and Notch-driven tumorigenesis in *Drosophila*. PLoS One 10: e0132987. 26207831 10.1371/journal.pone.0132987 PMC4514741

[bib18] Dollar G. L. Weber U. Mlodzik M. Sokol S. Y., 2005 Regulation of Lethal giant larvae by Dishevelled. Nature 437: 1376–1380. 16251968 10.1038/nature04116

[bib19] Dong W. Zhang X. Liu W. Chen Y. J. Huang J., 2015 A conserved polybasic domain mediates plasma membrane targeting of Lgl and its regulation by hypoxia. J. Cell Biol. 211: 273–286. 26483556 10.1083/jcb.201503067 PMC4621827

[bib20] Elsum I. A. Humbert P. O., 2013 Localization, not important in all tumor-suppressing properties: a lesson learnt from Scribble. Cells Tissues Organs 198: 1–11. 23774808 10.1159/000348423

[bib21] Enomoto M. Igaki T., 2013 Src controls tumorigenesis via JNK-dependent regulation of the Hippo pathway in *Drosophila*. EMBO Rep. 14: 65–72. 23196366 10.1038/embor.2012.185 PMC3537139

[bib22] Erneux C. Ghosh S. Ramos A. R. Edimo W. E., 2016 New functions of the inositol polyphosphate 5-phosphatases in cancer. Curr. Pharm. Des. 22: 2309–2314. 26916021 10.2174/1381612822666160226132512

[bib23] Fletcher G. C. Lucas E. P. Brain R. Tournier A. Thompson B. J., 2012 Positive feedback and mutual antagonism combine to polarize crumbs in the *Drosophila* follicle cell epithelium. Curr. Biol. 22: 1116–1122. 22658591 10.1016/j.cub.2012.04.020

[bib24] Friedman A. A. Tucker G. Singh R. Yan D. Vinayagam A., 2011 Proteomic and functional genomic landscape of receptor tyrosine kinase and Ras to extracellular signal-regulated kinase signaling. Sci. Signal. 4: rs10. 22028469 10.1126/scisignal.2002029 PMC3439136

[bib25] Grzeschik N. A. Knust E., 2005 IrreC/rst-mediated cell sorting during *Drosophila* pupal eye development depends on proper localisation of DE-cadherin. Development 132: 2035–2045. 15788453 10.1242/dev.01800

[bib26] Grzeschik N. A. Amin N. Secombe J. Brumby A. M. Richardson H. E., 2007 Abnormalities in cell proliferation and apico-basal cell polarity are separable in *Drosophila lgl* mutant clones in the developing eye. Dev. Biol. 311: 106–123. 17870065 10.1016/j.ydbio.2007.08.025 PMC2974846

[bib27] Grzeschik N. A. Parsons L. M. Allott M. L. Harvey K. F. Richardson H. E., 2010 a Lgl, aPKC, and crumbs regulate the Salvador/Warts/Hippo pathway through two distinct mechanisms. Curr. Biol. 20: 573–581. 20362447 10.1016/j.cub.2010.01.055

[bib28] Grzeschik N. A. Parsons L. M. Richardson H. E., 2010 b Lgl, the SWH pathway and tumorigenesis: it’s a matter of context & competition! Cell Cycle 9: 3202–3212. 20724829 10.4161/cc.9.16.12633

[bib29] Guruharsha K. G. Rual J. F. Zhai B. Mintseris J. Vaidya P., 2011 A protein complex network of *Drosophila melanogaster*. Cell 147: 690–703. 22036573 10.1016/j.cell.2011.08.047 PMC3319048

[bib30] Hariharan I. K. Bilder D., 2006 Regulation of imaginal disc growth by tumor-suppressor genes in *Drosophila*. Annu. Rev. Genet. 40: 335–361. 16872256 10.1146/annurev.genet.39.073003.100738

[bib31] Harvey K. Tapon N., 2007 The Salvador–Warts–Hippo pathway—an emerging tumour-suppressor network. Nat. Rev. Cancer 7: 182–191. 17318211 10.1038/nrc2070

[bib32] Hirabayashi S. Cagan R. L., 2015 Salt-inducible kinases mediate nutrient-sensing to link dietary sugar and tumorigenesis in *Drosophila*. eLife 4: e08501. 26573956 10.7554/eLife.08501 PMC4643014

[bib33] Huang H. Du G. Chen H. Liang X. Li C., 2011 *Drosophila* Smt3 negatively regulates JNK signaling through sequestering Hipk in the nucleus. Development 138: 2477–2485. 21561986 10.1242/dev.061770

[bib34] Huttlin E. L. Ting L. Bruckner R. J. Gebreab F. Gygi M. P., 2015 The BioPlex network: a systematic exploration of the human interactome. Cell 162: 425–440. 26186194 10.1016/j.cell.2015.06.043 PMC4617211

[bib35] Ile K. E. Tripathy R. Goldfinger V. Renault A. D., 2012 Wunen, a *Drosophila* lipid phosphate phosphatase, is required for septate junction-mediated barrier function. Development 139: 2535–2546. 22675212 10.1242/dev.077289

[bib36] Johnson K. Grawe F. Grzeschik N. Knust E., 2002 *Drosophila* crumbs is required to inhibit light-induced photoreceptor degeneration. Curr. Biol. 12: 1675–1680. 12361571 10.1016/s0960-9822(02)01180-6

[bib37] Kagey J. D. Brown J. A. Moberg K. H., 2012 Regulation of Yorkie activity in *Drosophila* imaginal discs by the Hedgehog receptor gene *patched*. Mech. Dev. 129: 339–349. 22705500 10.1016/j.mod.2012.05.007 PMC3668547

[bib38] Kaplan N. A. Tolwinski N. S., 2010 Spatially defined Dsh-Lgl interaction contributes to directional tissue morphogenesis. J. Cell Sci. 123: 3157–3165 20736316 10.1242/jcs.069898

[bib39] Kaplan N. A. Liu X. Tolwinski N. S., 2009 Epithelial polarity: interactions between junctions and apical-basal machinery. Genetics 183: 897–904. 19737741 10.1534/genetics.109.108878 PMC2778985

[bib40] Kaplan N. A. Colosimo P. F. Liu X. Tolwinski N. S., 2011 Complex interactions between GSK3 and aPKC in Drosophila embryonic epithelial morphogenesis. PLoS One 6: e18616. 21483653 10.1371/journal.pone.0018616 PMC3071738

[bib41] Kiger J. A. Jr. O’Shea C., 2001 Genetic evidence for a protein kinase A/cubitus interruptus complex that facilitates processing of cubitus interruptus in Drosophila. Genetics 158: 1157–1166. 11454764 10.1093/genetics/158.3.1157 PMC1461713

[bib42] Klebes A. Knust E., 2000 A conserved motif in Crumbs is required for E-cadherin localisation and zonula adherens formation in *Drosophila*. Curr. Biol. 10: 76–85. 10662667 10.1016/s0960-9822(99)00277-8

[bib43] Klein T. J. Jenny A. Djiane A. Mlodzik M., 2006 CKIɛ/*discs overgrown* promotes both Wnt-Fz/β-catenin and Fz/PCP signaling in *Drosophila*. Curr. Biol. 16: 1337–1343. 16824922 10.1016/j.cub.2006.06.030

[bib44] Lee W. Andrews B. C. Faust M. Walldorf U. Verheyen E. M., 2009 Hipk is an essential protein that promotes Notch signal transduction in the *Drosophila* eye by inhibition of the global co-repressor Groucho. Dev. Biol. 325: 263–272. 19013449 10.1016/j.ydbio.2008.10.029

[bib45] Li W. Ohlmeyer J. T. Lane M. E. Kalderon D., 1995 Function of protein kinase A in hedgehog signal transduction and Drosophila imaginal disc development. Cell 80: 553–562. 7867063 10.1016/0092-8674(95)90509-x

[bib46] Ling C. Zheng Y. Yin F. Yu J. Huang J., 2010 The apical transmembrane protein crumbs functions as a tumor suppressor that regulates Hippo signaling by binding to expanded. Proc. Natl. Acad. Sci. USA 107: 10532–10537. 20498073 10.1073/pnas.1004279107 PMC2890787

[bib47] Liu Y. Bankaitis V. A., 2010 Phosphoinositide phosphatases in cell biology and disease. Prog. Lipid Res. 49: 201–217. 20043944 10.1016/j.plipres.2009.12.001 PMC2873057

[bib48] Ma X. Shao Y. Zheng H. Li M. Li W., 2013 Src42A modulates tumor invasion and cell death via Ben/dUev1a-mediated JNK activation in *Drosophila*. Cell Death Dis. 4: e864. 24136228 10.1038/cddis.2013.392 PMC3920939

[bib49] McCaffrey L. M. Macara I. G., 2011 Epithelial organization, cell polarity and tumorigenesis. Trends Cell Biol. 21: 727–735. 21782440 10.1016/j.tcb.2011.06.005

[bib50] Milton C. C. Zhang X. Albanese N. O. Harvey K. F., 2010 Differential requirement of Salvador-Warts-Hippo pathway members for organ size control in *Drosophila melanogaster*. Development 137: 735–743. 20110315 10.1242/dev.042309

[bib51] Morrison D. K. Murakami M. S. Cleghon V., 2000 Protein kinases and phosphatases in the *Drosophila* genome. J. Cell Biol. 150: F57–F62. 10908587 10.1083/jcb.150.2.f57 PMC2180215

[bib52] Ogawa H. Ohta N. Moon W. Matsuzaki F., 2009 Protein phosphatase 2A negatively regulates aPKC signaling by modulating phosphorylation of Par-6 in *Drosophila* neuroblast asymmetric divisions. J. Cell Sci. 122: 3242–3249. 19690050 10.1242/jcs.050955

[bib53] Oh H. Irvine K. D., 2011 Cooperative regulation of growth by Yorkie and Mad through *bantam*. Dev. Cell 20: 109–122. 21238929 10.1016/j.devcel.2010.12.002 PMC3033745

[bib54] Parker J. Struhl G., 2015 Scaling the *Drosophila* wing: TOR-dependent target gene access by the Hippo pathway transducer Yorkie. PLoS Biol. 13: e1002274. 26474042 10.1371/journal.pbio.1002274 PMC4608745

[bib55] Parsons L. M. Grzeschik N. A. Allott M. L. Richardson H. E., 2010 Lgl/aPKC and Crb regulate the Salvador/Warts/Hippo pathway. Fly (Austin) 4: 288–293. 20798605 10.4161/fly.4.4.13116 PMC3174480

[bib56] Parsons L. M. Portela M. Grzeschik N. Richardson H. E., 2014 a Lgl regulates Notch signaling via endocytosis, independently of the apical aPKC-Par6-Baz polarity complex. Curr. Biol. 24: 2073–2084. 25220057 10.1016/j.cub.2014.07.075

[bib57] Parsons L. M. Grzeschik N. A. Richardson H. E., 2014 b lgl regulates the Hippo pathway independently of Fat/Dachs, Kibra/Expanded/Merlin and dRASSF/dSTRIPAK. Cancers (Basel) 6: 879–896. 24743776 10.3390/cancers6020879 PMC4074808

[bib58] Plant P. J. Fawcett J. P. Lin D. C. Holdorf A. D. Binns K., 2003 A polarity complex of mPar-6 and atypical PKC binds, phosphorylates and regulates mammalian Lgl. Nat. Cell Biol. 5: 301–308. 12629547 10.1038/ncb948

[bib59] Poon C. L. Lin J. I. Zhang X. Harvey K. F., 2011 The sterile 20-like kinase Tao-1 controls tissue growth by regulating the Salvador-Warts-Hippo pathway. Dev. Cell 21: 896–906. 22075148 10.1016/j.devcel.2011.09.012

[bib60] Portela M. Parsons L. M. Grzeschik N. A. Richardson H. E., 2015 Regulation of Notch signaling and endocytosis by the Lgl neoplastic tumor suppressor. Cell Cycle 14: 1496–1506. 25789785 10.1080/15384101.2015.1026515 PMC4613855

[bib61] Read R. D. Bach E. A. Cagan R. L., 2004 *Drosophila* C-terminal Src kinase negatively regulates organ growth and cell proliferation through inhibition of the Src, Jun N-terminal kinase, and STAT pathways. Mol. Cell. Biol. 24: 6676–6689. 15254235 10.1128/MCB.24.15.6676-6689.2004 PMC444864

[bib62] Reddy B. V. Irvine K. D., 2013 Regulation of Hippo signaling by EGFR-MAPK signaling through Ajuba family proteins. Dev. Cell 24: 459–471. 23484853 10.1016/j.devcel.2013.01.020 PMC3624988

[bib63] Richardson E. C. Pichaud F., 2010 Crumbs is required to achieve proper organ size control during *Drosophila* head development. Development 137: 641–650. 20110329 10.1242/dev.041913 PMC2827617

[bib64] Richardson H. E. Portela M., 2017 Tissue growth and tumorigenesis in *Drosophila*: cell polarity and the Hippo pathway. Curr. Opin. Cell Biol. 48: 1–9. 28364663 10.1016/j.ceb.2017.03.006

[bib65] Robinson B. S. Huang J. Hong Y. Moberg K. H., 2010 Crumbs regulates Salvador/Warts/Hippo signaling in *Drosophila* via the FERM-domain protein expanded. Curr. Biol. 20: 582–590. 20362445 10.1016/j.cub.2010.03.019 PMC2855393

[bib66] Rogulja D. Rauskolb C. Irvine K. D., 2008 Morphogen control of wing growth through the fat signaling pathway. Dev. Cell 15: 309–321. 18694569 10.1016/j.devcel.2008.06.003 PMC2613447

[bib67] Shackelford D. B. Shaw R. J., 2009 The LKB1-AMPK pathway: metabolism and growth control in tumour suppression. Nat. Rev. Cancer 9: 563–575. 19629071 10.1038/nrc2676 PMC2756045

[bib68] Shewan A. Eastburn D. J. Mostov K., 2011 Phosphoinositides in cell architecture. Cold Spring Harb. Perspect. Biol. 3: a004796. 21576256 10.1101/cshperspect.a004796 PMC3140688

[bib69] Shi Q. Li S. Li S. Jiang A. Chen Y., 2014 Hedgehog-induced phosphorylation by CK1 sustains the activity of Ci/Gli activator. Proc. Natl. Acad. Sci. USA 111: E5651–E5660. 25512501 10.1073/pnas.1416652111 PMC4284548

[bib70] Sopko R. Silva E. Clayton L. Gardano L. Barrios-Rodiles M., 2009 Phosphorylation of the tumor suppressor fat is regulated by its ligand Dachsous and the kinase discs overgrown. Curr. Biol. 19: 1112–1117. 19540118 10.1016/j.cub.2009.05.049 PMC2851237

[bib71] Sopko R. Foos M. Vinayagam A. Zhai B. Binari R., 2014 Combining genetic perturbations and proteomics to examine kinase-phosphatase networks in *Drosophila* embryos. Dev. Cell 31: 114–127. 25284370 10.1016/j.devcel.2014.07.027 PMC4208667

[bib72] Sotillos S. Diaz-Meco M. T. Caminero E. Moscat J. Campuzano S., 2004 DaPKC-dependent phosphorylation of crumbs is required for epithelial cell polarity in *Drosophila*. J. Cell Biol. 166: 549–557. 15302858 10.1083/jcb.200311031 PMC2172211

[bib73] Sun G. Irvine K. D., 2011 Regulation of Hippo signaling by Jun kinase signaling during compensatory cell proliferation and regeneration, and in neoplastic tumors. Dev. Biol. 350: 139–151. 21145886 10.1016/j.ydbio.2010.11.036 PMC3038240

[bib74] Swarup S. Verheyen E. M., 2011 *Drosophila* homeodomain-interacting protein kinase inhibits the Skp1-Cul1-F-box E3 ligase complex to dually promote Wingless and Hedgehog signaling. Proc. Natl. Acad. Sci. USA 108: 9887–9892. 21628596 10.1073/pnas.1017548108 PMC3116437

[bib75] Swarup S. Pradhan-Sundd T. Verheyen E. M., 2015 Genome-wide identification of phospho-regulators of Wnt signaling in *Drosophila*. Development 142: 1502–1515. 25852200 10.1242/dev.116715

[bib76] Tepass U., 2012 The apical polarity protein network in *Drosophila* epithelial cells: regulation of polarity, junctions, morphogenesis, cell growth, and survival. Annu. Rev. Cell Dev. Biol. 28: 655–685. 22881460 10.1146/annurev-cellbio-092910-154033

[bib77] Tyler D. M. Baker N. E., 2007 *Expanded* and *fat* regulate growth and differentiation in the *Drosophila* eye through multiple signaling pathways. Dev. Biol. 305: 187–201. 17359963 10.1016/j.ydbio.2007.02.004 PMC2075468

[bib78] Vereshchagina N. Ramel M. C. Bitoun E. Wilson C., 2008 The protein phosphatase PP2A-B’ subunit Widerborst is a negative regulator of cytoplasmic activated Akt and lipid metabolism in *Drosophila*. J. Cell Sci. 121: 3383–3392. 18827008 10.1242/jcs.035220

[bib79] Vissers J. H. Manning S. A. Kulkarni A. Harvey K. F., 2016 A *Drosophila* RNAi library modulates Hippo pathway-dependent tissue growth. Nat. Commun. 7: 10368. 26758424 10.1038/ncomms10368 PMC4735554

[bib80] Wang B. Moya N. Niessen S. Hoover H. Mihaylova M. M., 2011 A hormone-dependent module regulating energy balance. Cell 145: 596–606. 21565616 10.1016/j.cell.2011.04.013 PMC3129781

[bib81] Wehr M. C. Holder M. V. Gailite I. Saunders R. E. Maile T. M., 2013 Salt-inducible kinases regulate growth through the Hippo signalling pathway in *Drosophila*. Nat. Cell Biol. 15: 61–71. 23263283 10.1038/ncb2658 PMC3749438

[bib82] Yu J. S. Cui W., 2016 Proliferation, survival and metabolism: the role of PI3K/AKT/mTOR signalling in pluripotency and cell fate determination. Development 143: 3050–3060. 27578176 10.1242/dev.137075

[bib83] Zecca M. Struhl G., 2010 A feed-forward circuit linking wingless, fat-dachsous signaling, and the warts-hippo pathway to *Drosophila* wing growth. PLoS Biol. 8: e1000386. 20532238 10.1371/journal.pbio.1000386 PMC2879410

[bib84] Zeng W. Wharton K. A. Jr. Mack J. A. Wang K. Gadbaw M., 2000 Naked cuticle encodes an inducible antagonist of Wnt signalling. Nature 403: 789–795. 10693810 10.1038/35001615

[bib85] Zeng X. Singh S. R. Hou D. Hou S. X., 2010 Tumor suppressors Sav/Scrib and oncogene Ras regulate stem-cell transformation in adult *Drosophila* malpighian tubules. J. Cell. Physiol. 224: 766–774. 20432470 10.1002/jcp.22179 PMC3391499

[bib86] Zhu M. Xin T. Weng S. Gao Y. Zhang Y., 2010 Activation of JNK signaling links *lgl* mutations to disruption of the cell polarity and epithelial organization in *Drosophila* imaginal discs. Cell Res. 20: 242–245. 20066009 10.1038/cr.2010.2

