# Supplementary material for: A Kinome RNAi Screen in Drosophila Identifies Novel Genes Interacting with Lgl, aPKC, and Crb Cell Polarity Genes in Epithelial Tissues
Source: G3 (Bethesda). 2017 Jun 13;7(8):2497–509. doi: 10.1534/g3.117.043513 (PMC5555457; doi:10.1534/g3.117.043513)
Supplement: Supplementary file 1 [file 2497FileS1.pdf]

SUPPORTING INFORMATION

SUPPLEMENTAL FIGURES

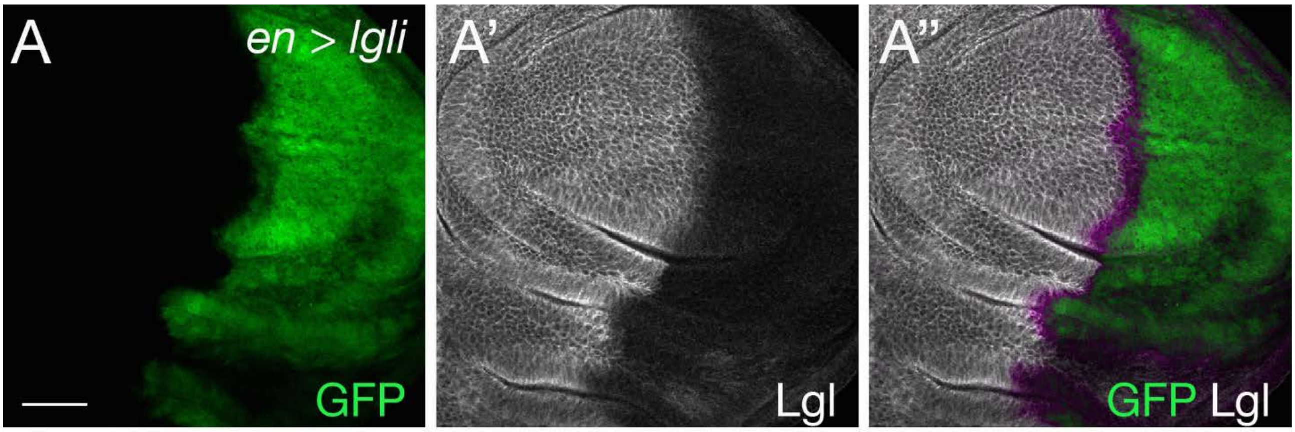

Parsons et al., Supp Figure S1

Figure S1. Validation of Lgl depletion

(A, A' and A'') Planar section of larval wing disc. Posterior is to the left. White scale bar represents 50μM.

(A) *en > GFP; lgli* wing disc posterior domain expression *en-GAL4* revealed by GFP+ tissue.

(A') Lgl staining shows reduced Lgl levels in posterior *en-GAL4* GFP+ tissue.

(A'') overlay of GFP+ tissue and Lgl staining confirms depletion of Lgl in posterior *en-GAL4* expression domain.

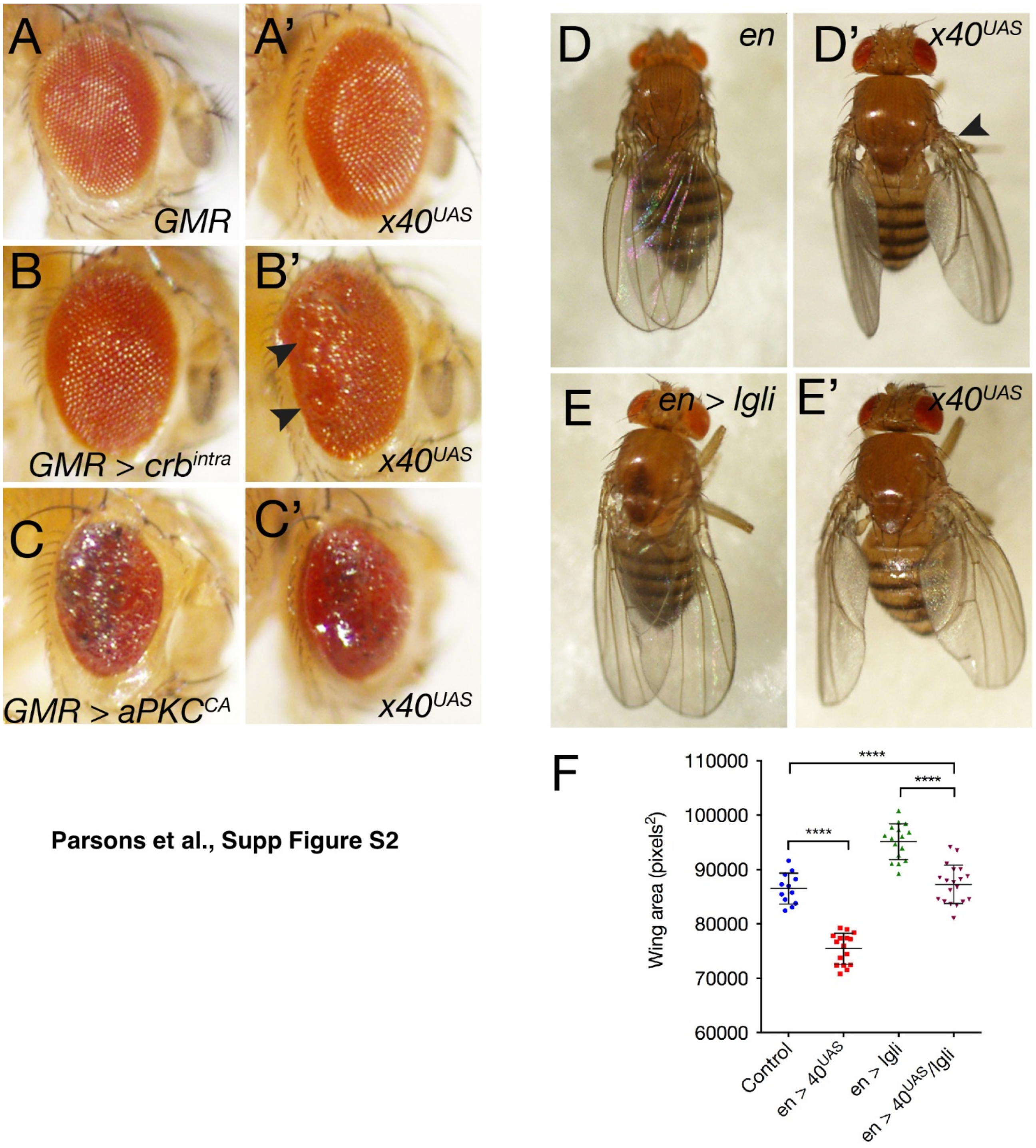

Parsons et al., Supp Figure S2

Figure S2. Cell polarity phenotype modification by ectopic *tio* expression

(A - C') Adult female eye images side views, posterior to the left.

(D-E') Adult female flies dorsal view.

(A) Control (*GMR - GAL4*) adult eyes displaying highly organized, regular, lattice like organization of ommatidia to form the adult retina which is unaffected by ectopic *tio* expression (A') 40^{UAS} (VDRC 60101).

(B) *GMR > crb*^{intra} adult eyes are slightly larger and rougher than control eyes. (B') ectopic *tio* expression 40^{UAS} (VDRC 60101) enhances the *GMR > crb*^{intra} tissue overgrowth phenotype (arrowheads).

(C) *GMR > aPKC*^{CA} adult eyes are smaller, areas of the retina show disruptions in retinal architecture and necrosis which is unaffected by ectopic *tio* expression (C') 40^{UAS}.

(D) Adult female *en - GAL4*, note wings are held straight back in line with the anterior/posterior axis. (D') Ectopic expression of *tio* (40^{UAS}) results in malformation of the hinge region (arrowhead), consequently the wings are held out and smaller.

(E) Adult female *en > lgli*, note wings are larger than control. (E') Ectopic expression of *tio* (40^{UAS}) results in malformation of the hinge region (arrowhead), consequently the wings are held out and smaller than *en > lgli*.

(F) Quantification of total wing area (D - E'). Results represent individual wings ± SD. ****$p < 0.0001$.

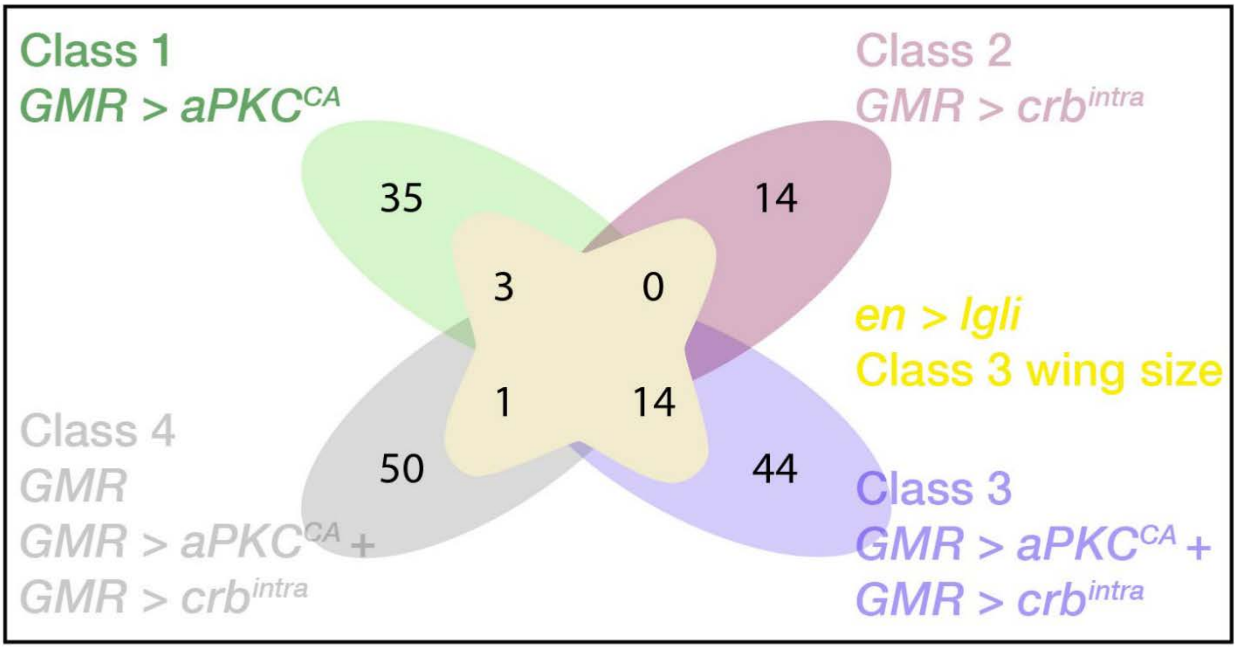

Parsons et al. Supp Figure S3

Figure S3. Intersection of interacting genes from the *en > lgli* wing size Classes 3.1, 3.3 and 3.4 with modifiers from the *GMR > aPKC*^{CA} and *GMR > crb*^{intra}

Venn diagram depicting the number of modifier genes that interacted with the *en > lgli* wing size Class Classes 1 - 3 from the adult *Drosophila* cell polarity eye screen. Yellow shading denotes *en > lgli* modifiers, that showed greater modification than with *en-GAL4*. Green shading denotes Class 1, *GMR > aPKC*^{CA}. Pink shading denotes Class 2, *GMR > crb*^{intra}. Central region denotes Class 3.1, 3.3 and 3.4 genes that modified *GMR > crb*^{intra}, *GMR > aPKC*^{CA}, *GMR > crb*^{intra} and *GMR > aPKC*^{CA} or all three eye phenotypes.

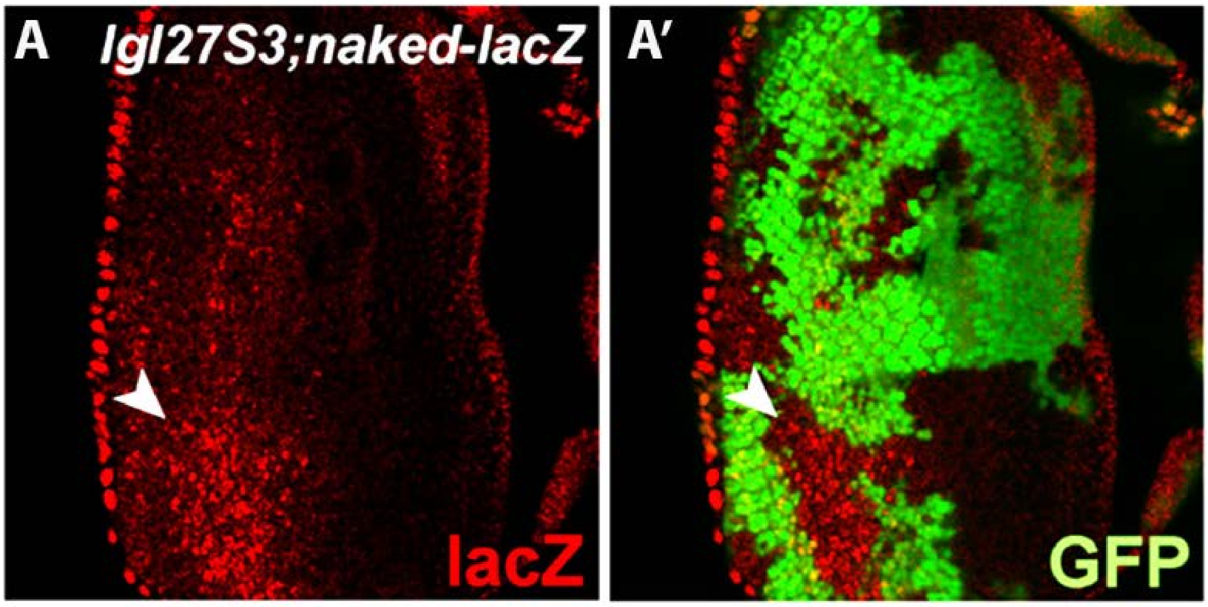

Parsons et al., Supp Figure S4

Figure S4 Wnt signalling is upregulated in *lgl* mutant tissue

(A and A') Planar section larval eye disc. Posterior is to the left. Nkd-lacZ is normally expressed in a gradient from the posterior to the anterior of the larval eye disc (Tyler and Baker, 2007). White arrowhead denotes *lgl*$^{-/-}$ mutant tissue with increased Nkd-β-galactosidase staining.

(A) *eyFLP*; *lgl*^{27S3}, *FRT40*/*Ubi-GFP*, *FRT40*; *nkd-lacZ/*+. GFP+ tissue is *wildtype*, GFP^{-} is *lgl*$^{-/-}$ mutant tissue. β-gal staining reveals higher levels of *nkd-lacZ* reporter gene expression in *lgl*$^{-/-}$ tissue compared to *wildtype*.

(A') Overlay of GFP+ tissue and β-gal staining confirms *lgl*$^{-/-}$ mutant tissue has higher levels of Wnt signalling than *wildtype* tissue.

SUPPLEMENTAL TABLES

Table S1. Classification of kinases and associated factors assayed in *Drosophila* adult eye cell polarity modifier screens

Tyrosine Kinase Like
STE (Homologs of yeast STE7, STE11 and STE20 genes)
Casein Kinase 1
Protein Kinase Like
CMGC (Named after a set of families (CDK, MAPK, GS3K and CLK)
AGC (Named after Protein Kinase A, G and C families)
Tyrosine Kinase
Receptor Guanylate Cyclase
Calcium and Calmodulin regulated Kinase
Atypical
Other
Unclassified (Diverse group of kinases and candidate kinases)
Kinase associated (Co factors such as cyclins)
Non protein Kinase (Lipid and sugar kinases)

CG	Gene	Gene Name	Gene Group	Gene Family	Human ortholog(s)	RNAi line
CG10033	DG2, for	foraging	AGC	PKG	PRKG1 PRKG2	38319
CG10079	Egfr	Epidermal growth factor receptor	TK	EGFR	ERB2 ERB3 ERB4 EGFR	107130

CG10082			Non-Protein Kinase	Lipid Kinase		103749 38326
CG10177			CAMK	DCAMKL		107848 38349
CG10244	Cad96Ca	Cadherin 96Ca	TK			1089
CG10260	PI4KIII□	Phosphatidylinositol 4-kinase III α	Non-Protein Kinase	Lipid Kinase	PI4KA	105614
CG10261	aPKC	atypical protein kinase C	AGC	PROTEIN KINASE C FAMILY	PRKCI PRKCZ	105624 2907
CG10498	cdc2c	cdc2	CMGC	CDK	CDK16 CDK14 CDK18	104959
CG10522	sti	sticky	AGC	DMPK	CIT	10522R-1
CG10539	S6k	RPS6-p70-protein kinase	AGC	RSK	RPS6KB1	104369 18126
CG10572	Cdk8	Cyclin-dependent kinase 8	CMCG	CDK	CDK8 CDK19	107187 31264
CG10579	Eip63E	Ecdysone-induced protein 63E	CMCG	CDK	CDK14 CDK16 CDK18	106824

CG10637	Nak	Numb-associated kinase	Other	NAK<br>Bud32	BMP2KL BMP2K AAK1 P53RK	35482
CG10702			Kinase associated			100842 3691
CG10776	wit	wishful thinking	TKL	STKR	BMPR2 ACVR2A TGFBR2 ACVR2B	103808
CG10895	lok	loki	CAMK	RAD53	CHEK2	44980 44981
CG10917	fj	four jointed	Atypical	FJX1	FJX1	6774
CG10951	niki	nimA like kinase	Other	NEK		100823
CG10967	Atg1	Autophagy specific gene 1	Other	ULK		16133
CG1098	Madm	MLF1-adaptor molecule	Other	NRBP	NRBP1<br>NRBP2	27346
CG1099	Dap160	Dynamin associated protein 160	Other		ITSN1<br>ITSN2	16158
CG1107	aux	auxillin	Other	NAK	DNAJC6<br>GAK	103426 16182

CG11221			Other	NKF-1		100163 42947
CG11228	hpo	hippo	STE	STE20	STK3<br>STK4	104169
CG11236			Other			38460
CG11420	png	pan gu	Other			31500
CG11486			Kinase associated		PAN3	106497
CG11489	srpk79D	serine-arginine protein kinase at 79D	CMCG	SRPK	SRPK1 SRPK2 SRPK3	102632
CG11533	Asator	Asator	CK1	TTBK	TTBK1<br>TTBK2	45120 45121
CG11621	Pi3K68D	Phosphotidylinositol 3 kinase 68D	Non Protein Kinase	Lipid Kinase	IK3C2G PIK3C2A PIK3C2B	16239
CG11660			Atypical	RIO	RIOK1<br>RIOK3	105395
CG11859			Atypical	RIO	RIOK2	47401

CG11870	Emk		STE	STK		10608816334
CG12066	Pka-C2	cAMP-dependent protein kinase 2	AGC	PKA	PRKACB	30658
CG12069	PKA-like		AGC	PKA		23719
CG12072	wts	warts	AGC	NDR	LATS1<br>LATS2	106174
CG1210	PdK1	Phosphoinositide-dependent kinase 1	AGC	PDK1	PDK1<br>PDK2	18736
CG12147	CKIa-like		CK1	CK1		101875 31658
CG1216	mri	mrityu	Kinase associated		KCTD20<br>BTBD10	101345
CG12170			Other		OXSM	31672
CG12244	lic	licorne	STE	STE7	MAP2K3 MAP2K4 MAP2K6	106822
CG1227	MPSK		Other	NAK		105610 38647
CG12306	polo	polo	Other	PLK	PLK1 PLK2 PLK3	20177

CG12559	rl	rolled	CMGC	MAPK	MAPK1 MAPK3	43123
CG13591	Ssl	Suppressor of Stellate like	CMGC		CSNK2B	17282
CG1362	cdc2rk	cdc2 related kinase	CMCG	CDK	CDC2L2	100299 32249
CG13688	Ipk2	Ipk2	PKL	IPK	IPMK	43824
CG13850	Ipk2	Ipk2	PKL	IPK	IPMK	100863
CG1389	tor	torso	Atypical	PIKK		101154 1016
CG14026	tkv	thickveins	TKL	STKR	ACVR1, BMPR1A, ACVR1C, BMPR1B, ACVRL1, ACVR1B	105834 862
CG14030	Bub1	Bub1 homologure	Other	BUB	BUB1	101096
CG14217	Tao-1	Tao	STE	STE20	TAOK1 TAOK2 TAOK3	17432
CG14226	dome	domeless	Other			106071
CG14305			CAMK	TSSK	TSSK1 TSSK2 TSSk3 TSSK4 TSSK6	17477
CG14396	Ret	Ret oncogene	TK	Ret	RET FGFR1 FGFR2	107648

					FGFR4	
CG14895	Pak3	Pak3	STE	STE20	PAK1 PAK2 PAK3	107260
CG14903			PTP	ClassIII	PTHRD1	105752
CG1495	CaMK1	Calcium/calmodulin-dependent protein kinase I	CAMK	CAMK1	PSKH2, CAMK4, CAMK1D, CAMK1D, PSKH1, PNCK, CAMK1	101380 1495R-1
CG14992	Ack	Activated Cdc42 kinase	TK	Ack	TNK1 TNK2	39857
CG15072	SIK3	Salt Inducible Kinase 3	CAMK	CAMKL	SIK1 SIK2 SIK3	39864
CG1511	Eph	Eph receptor tyrosine kinase	TK	Eph	EPHB6, EPHB3, EPHA1, EPHA10, EPHA4, EPHB2, EPHA8, EPHA2	4771
CG15224	CkIIβ	Casein kinase II beta subunit	CMGC	CK2	CSNK2B	106845 32377 32378
CG1578			Other	UK114 family	DPH6	41599
CG15793	Dsor1	Downstream of raf1	STE	STE7	MAP2K1, MAP2K2	107276 40026

CG1594	hop	hopscotch	TK	Jak		102830
CG1609	Gcn2	Gcn2	Other	PEK	EIFA2K4	103976
CG16708	Cerk	Ceramide Kinase	Non Protein Kinase	Lipid Kinase	CERK	101550
CG16910	key	Kenny	Kinase associated			100257
CG16973	msn	misshapen	STE	STE20	MINK1 MAP4K4 TNIK	16973R-1 (III)
CG17090	HIPK	homeodomain interacting protein kinase	CMGC	DYRK	HIPK1 HIPK2 HIPK3	32854
CG17161	grp	grapes	CAMK	CAMKL	CHEK1	12680
CG17216	KP78b		CAMK	STK	MARK1 MARK2 MARK3 MARK4	105265 51995
CG1725	dlg1	discs large 1	Kinase associated	MAGUK	DLG1 DLG2 DLG3 DLG4	41134 41136
CG17256	Nek2	Nek2	Other	NEK	NEK2 NEK6 NEK7	103408
CG17299	SNF4Aγ	SNF4/AMP-activated protein	PKL	PIPK	PRKAG1, PRKAG2,	17299R-1

		kinase gamma subunit			PRKAG3	
CG17342	MNK/LK6	MNK/LK6	CAMK	MAPKAPK	MKNK2	32885
CG17348	drl	Derailed	TK	Ryk like	AXL MERTK RYK TYRO	100039
CG17520	CKIIa	casein kinase IIα	CMGC	CK2	CSNK2A1 CSNK2A2	17520R-2 (III)
CG17559	dnt	doughnut on 2	TK	Ryk like	AXL MERTK RYK TYRO	106056
CG17596	S6kII	Ribosomal protein S6 kinase II	AGC	RSK	RPS6KA1 RPS6KA2 RPS6KA3 RPS6KA4 RPS6KA5 RPS6KA6	101451 5702
CG17603	Taf1	TBP-associated factor 1	Atypical	TAF1	TAF1 TAF1L	106119
CG17698	CAMKIIB		Other	CAMKK	CAMKK1 CAMKK2 CDNK1B	105884 35632
CG17998	Gprk2	G protein-coupled receptor kinase 2	AGC	GRK	GRK4 GRK5 GRK6	101463
CG18069	CaMKII	Calcium/calmodulin-dependent protein kinase II	CAMK	CAMK2	CAMK2A CAMK2B CAMK2D	38930

					CAMK2G	
CG18247	shark	SH2 ankyrin repeat kinase	TK	Syk		105706 25304
CG18255	Strn Mlck	Stretchin-Mlck	CAMK	MLCK	MYLK MYLK2 MYLK3 MYLK4	18255R-1 (III)
CG1830	PhKγ	Phosphorylase kinase γ	CAMK	PHK	PHKG2	33054
CG18402	InR	Insulin- ike receptor	TK	InsR	IGF1R	991
CG1848	LIMK1	LIM-kinase1	TKL	LISK	LIMK1 LIMK2	25343
CG18492	Tak1	TGF-β activated kinase 1	TKL	MLK	MAP3K7	101357 1388R-1 (II)
CG18582	mbt	mushroom bodies tiny	STE	STE20	PAK4 PAK6 PAK7	46043
CG18604	SIK3	Salt Inducible Kinase 3	STE	STK	SIK3	106268 20655
CG18734	Fur2	Furin 2	Kinase associated	Peptidase 8	FURIN PCSK1 PCSK2 PCSK4 PCSK5 PCSK6	101242
CG18741	DopR2	Dopamine 1-like receptor 2	Other	DOPAMINE RECEPTORS		105324
CG1891	Sax	saxophone	TKL	STKR	ACVR1, BMPR1A, ACVR1C, BMPR1B, ACVRL1	42457

CG1951			Other	SCY1	SCYL2	33431
CG1954	Pkc98E	Protein C kinase 98E	AGC	PKC	PRKCA PRKCB PRKCE<br>PRKCG<br>PRKCH<br>PRKCQ	33434
CG1973	yata	yata	Other	SCY1	SCYL2	19275
CG2028	CKIa	Casein kinase Iα	CK1	CK1	CSNK1A1, CSNK1D, CSNK1A1L, CSNK1E	13664
CG2048	dco	discs overgrown	CK1	CK1	CSNK1A1, CSNK1D, CSNK1A1L, CSNK1E	9241
CG2049	PRK2	PKN-related	AGC	PKN	PKN1 PKN2 PKN3	2055R-1
CG2056	c-SP33	Serine Protease Immune Response Integrator	Kinase associated	Peptidase S1	CELA3B<br>TPSG1……>60	107935
CG2087	PEK	pancreatic eIF-2α kinase	Other	PEK	EIF2AK3	16427
CG2210	awd	abnormal wing discs	Atypical	NDK	NME1 NME2 NME3 NME4	33198

CG2252	fs(l)h	female sterile (l) homeotic	Atypical		BRD2, BRD3, BRDT, BRD4	51227
CG2272	slpr	slipper	TKL	MLK	RP5-862P8.2, MLTK, MAP3K9, MAP3K10, MAP3K11	106449
CG2577	CKIa-like		CK1	CK1	CSNK1E, CSNK1A1, CSNK1A1L, CSNK1D	41693
CG2929	Pi4KII	Pi4KIIa	PKL	PIK	PI4K2A PI4K2B	25458
CG30021	metro	menage a trois	Non Protein Kinase	Nucleic Acid Kinase	MMP1 MMp2 MMp3 MMP4 MMP5 MMP7	29965
CG3008			Atypical	RIO	RIOK1 RIOk3	103828 52634
CG30274			Unclassified	guanido phophotransferase		33693
CG30295	Ipk1	Ipk1	PKL		IPPK	29406
CG30388	Magi		Unclassified	guanylate kinase activity	MAG1 MAG2 MAG3	41735
CG3051	SNF1A	AMP-activated protein kinase	CAMK	CAMKL	PRKAA1 PRKAA2	1827
CG3086	MAPK-Ak2	MAP kinase activated protein kinase-2	CAMK	MAPKAPK	MAPKAPK2 MAPKAPK3 MAPKAPK5	3170

CG31003	gskt		CMGC	GSK	GSK3A GSK3B	107429
CG3105	Pask	PAS kinase	CAMK	CAMKL	PASK	107025 25661
CG31097			Unclassified		AGPHD1	107652
CG31127	Wsck	Wsck	TK			1214
CG31140			Non Protein Kinase	Lipid Kinase	DGKQ	101347
CG31183			Kinase associated		NPR1 NPR2	858
CG31349	pyd	polychaetoid	Kinase associated		TJP1 TJP2 TJP3	38863
CG3135	shf	shifted	Unclassified	EGF like	WIF1A	14803
CG31421	TAKL1	Tak1-like 1	TLK		MAP3K7	25760
CG31431			Unclassified	IgG like		104697
CG31643	CG31643		Atypical		FASTKD1, TBRG4, FASTKD3, FASTK, FASTKD3	31643 R-1
CG3172	twf	twinflin	TK		TWF1 TWF2	25817
CG31873			Non Protein Kinase	Lipid Kinase	FLJ10842	105621

CG32019	bt	bent	CAMK		MYLK MYLK2 MYLK3 MYLK4	46252
CG32134	btl	breathless	TK		RET<br>FGFR1 FGFR2 FGFR3 FGFR4	947
CG3216			RGC	RGC	NPR1<br>NPR2	107985
CG32417	Myt1	Myt1	AGC		PKMYT1	105157
CG32484	Sk2	Sphingosine kinase 2	Non Protein Kinase	Lipid Kinase		101018
CG32703	ERK7	Extracellularly regulated kinase 7	CMGC		MAPK15	2309R-1 (III)
CG32717	sdt		Unclassified	guanylate kinase activity	MMP1<br>MMP2 MMMP3<br>CASK	100685
CG32742	l(1)G0148		Other		CDC7	104902
CG32743	nonC	no-on-and-no-off transient C	PKL		SMG1	4549 R-1

CG3277			TK	VEGFR		108052
CG32944			CAMK		STK32A STK32B STK32C	103642 10532R-1 (II)
CG3319	Cdk7	Cyclin-dependent kinase 7	CMGC	CDK	CDK7 CDK20	103413
CG3324	Pkg21D	21D	AGC	PKG		103513 34594
CG33338	p38c	P38c	CMGC	MAPK	MAPK11 MAPK12 MAPK13 MAPK14	105173
CG33519	Unc-89	Unc-89	Unclassified			106267
CG33554	Nipped-A	Nipped-A	Atypical	PIKK	TRRAP	2905 R-3
CG33671			Non Protein Kinase	Carbohydrate Kinase	MVK	49772
CG3400	Pfrx	6-phosphofructo-2-kinase	Non Protein Kinase	Carbohydrate Kinase	PFKFB1 PFKFB2 PFKFB3 PFKFB4	25958
CG34123	Trpm	Transient receptor potential cation channel, subfamily M ortholog	Other	Transient receptor potential channels	TRPM1 TRPM3 TRPM6 TRPM7	107537
CG34344	rdgA	retinal degeneration A	Non Protein Kinase	Lipid Kinase	DGKZ DGKZ1	103806
CG34357			RGC		NPR1 NPR2	105185
CG34359	IP3K2	Inositol 1,4,5-triphosphate	Non Protein Kinase	Lipid Kinase	ITPK1	102772

		kinase 2				
CG34361	Dgk	Diacyl glycerol kinase	Non Protein Kinase	Lipid Kinase	DGKA DGKB DGKG	105753
CG34380			TK		DDR1 DDR2	31640 R-1
CG34384			Non Protein Kinase	Lipid Kinase	DGKD DGKH	102481
CG34412	tlk		Other		TLK1<br>TLK2	105732 46425
CG3608			Atypical	ABC1	ADCK1	106695
CG3682	PIP5K59B	PIP5K59B	PKL		PSMD4 PIP5K1A<br>PIP5K1B<br>PIP5K1C	47027
CG3724	Pgd	Phosphogluconate dehydrogenase	Other	dehydrogenase	PGD	100269
CG3837			Kinase Associated			105549
CG3915	Drl-2	derailed 2	TK	Ryk	AXL MERTK RYK TYRO3	102192
CG3969	Ack-like	Activated Cdc42 kinase-like	TK			100169
CG4007	Nrk	Neurospecific receptor kinase	TK	Musk	MUSK1 ROR1 ROR2	103804

CG4012	Gek	Genghis Kahn	AGC	DMPK	CDC42BPA CDC42BPB CDC42BPG	28367
CG4041			Other	TBCK	TBCK	34780
CG4141	Pi3K92E	Pi3K92E	PKL		PIK3CA PIK3CB PIK3CD PIK3CG	38986
CG4252	mei-41	meiotic 41	Atypical	PIKK	ATR	103624 11251
CG4268	Pitslre	Pitslre	CMGC	CDK	CDC2L2	107303
CG4290	SIK2	Salt Inducible Kinase 2	CAMK	CAMKL	SIK1 SIK2 SIK3	103739 26496
CG4379	Pka-C1	cAMP-dependent protein kinase 1	AGC	PKA	PRKACA PRKACB PRKACG PRKX	101524
CG4488	wee	wee	Other	WEE	WEE1 WEE2	106329
CG4523	dPINK1	PTEN-induced putative kinase 1	Other	NKF2	PINK1	21860
CG4527	slik	Sterile20-like kinase	STE	STE20	STK10	43783
CG4551	smi35A	smell impaired 35A	CMGC	DYRK	DYRK1A DYRK1B DYRK2	101376

					DYRK3 DYRK4	
CG4583	Ire-1	Inositol-requiring enzyme-1	Other	IRE	ERN1 ERN2	39561
CG4629			CAMK	CAMKL	NIM1	26574
CG4803	Takl2	Takl-like 2	TKL	MLK	MAP3K7	104701
CG4839			AGC	PKG	PRKG1 PRKG2	100999 26641
CG4926	Ror	Ror	TK	Ror	ROR1 ROR2 MUSK	932
CG4945			Other	NFK1		24683
CG4965			Other	NFK1		104147 46064
CG5072	Cdk4	Cyclin-dependent kinase 4	CMGC	CDK1	CDK4 CDK6	40576
CG5092	Tor	Target of rapamycin	Atypical	PIKK	MTOR	5092R-2
CG5125	ninaC	neither inactivation nor afterpotential C	STE	STE20		27359
CG5169	GckIII	Germinal centre kinase III	STE	STE20	MST4 STK24 STK25	107158 49558
CG5179	Cdk9	Cyclin-dependent kinase 9	CMGC	CDK	CDK4 CDK6	103561 30448
CG5182	Pk34A	Pk34A	CMGC	GSK		101146 27368
CG5363	cdc2	cdc2	CMGC	CDK	CDK1 CDK2 CDK3	106130 41838

CG5373	Pi3K59F	Phosphotidylinositol 3 kinase 59F	Non Protein Kinase	Lipid Kinase	PI3KC3	100296
CG5387	Cdk5a	Cdk5 activator-like protein	Kinase Associated		CDK5R1	34990
CG5408	trbl	tribbles	CAMK	Trbl	TRIB1 TRIB2 TRIB3	106774
CG5475	Mpk2	Mpk2	CMGC	MAPK	MAPK9 MAPK10	52277
CG5483	Lrrk	Leucine-rich repeat kinase	TKL	LRRK	LRRK1 LRRK2	105630
CG5680	bsk	basket	CMGC	MAPK	MAPK9 MAPK10	34138
CG5725	fbl	fumble	Non Protein Kinase	Lipid Kinase	PANK1 PANK2 PANK3	101437
CG5757			Non Protein Kinase	Nucleic Acid Kinase	DTYMK	27504
CG5790			Other	CDC7	CDC7	45045
CG5974	pll	pelle	TKL	IRAK	IRAK2	103774
CG6027	cdi	center divider	TKL	LISK	TESK1	43633
CG6114	Sad	synapses of amphids defective	CAMK	CAMKL	BRSK1 BRSK2	100717 22225
CG6214	MRP	Multidrug-Resistance like Protein 1	Kinase Associated		ABCC1 ABCC2 ABCC3	105419

					ABCC4	
CG6292	CycT	cyclinT	CMGC	CDK	CCNT1 CCNT2	103387
CG6297	JIL-1	JIL-1	AGC	RSK	RPS6KA1 to RPS6KA6	107001
CG6355	fab1	fab2	PKL		PIKFYVE	27591
CG6386	ball	ballchen	CK1	VRK	VRK1<br>VRK2<br>VRK3	48980
CG6498	dop	drop out	AGC	MASR	MAST1<br>MAST2<br>MAST3<br>MAST4	35101
CG6518	inaC	no inactivation after potential C	AGC	PKC	PRKCA<br>PRKCB<br>PRKCE<br>PRKCH<br>PRCKQ	101719 2895

CG6535	tefu	telomerase fusion	Atypical	PIKK	ATM	22502
CG6551	fu	fused	Other	ULK	STK36	27662
CG6620	ial	Ipll-aurora-like kinase	Other	AUR	AURKA AURKB AURKC	104051
CG6622	Pkc53E	Protein C Kinase 53E	AGC	PKC	PRKCA PRKCB PRKCE PRKCG PRKCH PRKCQ	27696
CG6703	CASK	CASK ortholog	CAMK	MAGUK		104793 34184
CG6715	KP78A	KP78A	CAMK	CAMKL	MARK1 MARK2 MARK3 MARK4	51616
CG6772	Slob	Slowpoke binding protein	Other	Slob		100987
CG6800			CMGC	CDK	CDK7 CDK20	104255
CG6875	asp	abnormal spindle	Kinase Associated		ASPM	2910
CG6963	gish,	gilgamesh	CK1	CK1	CSNK1G1 CSNK1G2 CSNK1G3	106826 26003
CG7001	S6KL	S6 Kinase Like	AGC	RSKR		102179

CG7004	fwd	four wheel drive	PKL		PIK4B	27785
CG7028	PRP4	PRP4	CMGC	DYRK	PRPF4B	107042 27808
CG7094			CK1	CK1	CSNK1A1 CSNK1A1L CSNK1D<br><br>CSNK1E	27843
CG7097	hppy	happyhour	STE	STE20	MAP4K1 MAP4K2 MAP4K3 MAP4K5	103580 35166
CG7125	PKD	Protein Kinase D	CAMK	PKD	PRKD1 PRKD2 PRKD3	106255
CG7156			AGC	RSKL		26035
CG7177	Wnk	WNK homolog	Other	WNK	WNK1 WNK2 WNK3 WNK4	106928
CG7186	Sak	SAK Kinase	Other	PLK	PLK4	105102
CG7207	cert	ceramide transfer protein	Kinase Associated		COL4A3BP	103563
CG7223	htl	heartless	TK	FGF	FGFR2	6692
CG7393	p38b	p38b	CMGC	MAPK	MAPK11	7393R-3 (III)
CG7497			Other			106421 9374
CG7525	Tie	Tie like receptor tyrosine kinase	TK		TIE1	26879

CG7597	Cdk12	Cdk12	CGMC	Cdk	Cdk9 Cdk12 Cdk13	25508
CG7616			Atypical	ABC1	ADCK5	104663
CG7643	ald	altered disjunction	Other	TTK	TTK	7643 R-1
CG7693	fray	frayed	STE	STE20	OXSR1	106919 7693-R-2 (III)
CG7717	Mekk1	Mekk1	STE	STE11	MAP3K4	25528
CG7719	gwl	greatwall	AGC	MAST	MASTL	21046
CG7838	BubR1	Bub related kinase	Other	BUB	BUB1B	26109
CG7873	Src42A	Src oncogene at 42	TK	Src	HCK LCK<br>FRK SRC BLK YES FGR FYN LYN	100708
CG7892	nmo	nemo	CMGC	MAAPK	NLK	101545 3002
CG7904	put	punt	TKL	STKR	ACVR2A ACVR2B BMPR2 TGFBR2	107071 37279
CG8049	Btk29A	Btk kinase family at 29A	TK	Tec	BMX BTX ITK TEC TXK	106962
CG8057	alc	alcorn	Non Protein Kinase	Lipid Kinase	GK GK2	104489
CG8173			Other	TOPK	PBK	35845

CG8174	SRPK	SRPK	CMGC	SRPK	SRPK1 SRPK2 SRPK3	26933
CG8201	par1	par1	CAMK	CAMKL	MARK1 MARK2 MARK3 MARK4	52556
CG8203	Cdk5	Cyclin dependent kinase 5	CMGC	CDK	CDK5	104491 35855
CG8222	Pvr	PDGF and VEGF receptor related	TK	VEGFR	VEGFR1 VEGFR2 VEGFR3	105353 976
CG8224	babo	baboon	TKL	STKR	ACR1 ACR1B ACVR1C ACVRL1 BMPR1A BMPR1B	106092 853
CG8250	Alk	Alk	TK	ALK	ALK LTK ROS1	107083
CG8351	Tcp-1-eta1	Tcp-1-eta1	Kinase Associated		CCT7	28895
CG8485			CAMK	CAMKL	SNRK	35939
CG8565			CMGC	SRPK	SPRK1 SPRK2 SPRK3	100449 8565R-2 (III)
CG8637	trc	tricornered	AGC	NDR	STK38	107923 35988

CG8657	Dgk	Diacy glycerol kinase	Non Protein Kinase	Lipid Kinase		4659
CG8726			Other	Slob	PXK	40719
CG8742	Gyc76C	Guanylyl cyclase at 76C	Other	Putative Pseudokinase	NPR1 NPR2	106525
CG8767	mos	mos	Other	MOS	MOS	36531
CG8789	wnd	wallenda	TKL	MLK	MAP3K12 MAP3K13	103410
CG8866			Other	ULK	ULK3	103725
CG8874	Fps85D	Fps oncogene analog	TK	Fer	FER	107266 36053
CG8878			CK1	VRK		100985 28970
CG8914	CkIIbeta2	Caesin kinase II b2 subunit	CMGC		CSNK2B	102633 26915
CG8967	otk	off track	TK	CCK4	PTK7	104688
CG9222			CAMK	TSSK	TSSK1B TSSK2 TSSK4 TSSK6	104259

CG9358	Phk-3	Pherokine	Non Protein Kinase	Lipid Kinase		106938
CG9738	Mkk4	MAP kinase kinase 4	STE	STE7	MAP2K3 MAP2K4 MAP2K6	26929
CG9774	rok	Rho kinase	AGC	DMPK	ROCK1 ROCK2	3793
CG9962			CK1	CK1		36473
CG9985	sktl	skittles	PKL		PIP5K1A PIP5K1B PIP5K1C PSMD4	101624 6229

Kinases and kinase-associated factors assayed in *Drosophila* adult eye cell polarity modifier screens (sorted by CG number) encoding either kinases or associated factors for which *UAS*-controlled inverted repeat strains were tested. Kinase group and family are indicated (see color-coded chart below), as well as human orthologs. RNAi line refers to VDRC number and those numbers denoted with R were obtained from NIG (see *Drosophila* stocks, Materials and Methods for further information).

Table S2. Classification of phosphatases and phosphatase-associated factors assayed in *Drosophila* adult eye cell polarity modifier screens

Phosphoprotein Phosphatase (PPP)
Protein Phosphatase Mg+/Mn+ dependent (PPM)
Classical Protein Tyrosine Phosphatase
ClassI/Dual Specificity Phosphatase (DSP)
Class III protein Tyrosine Phosphatase
Factor IIF interacting CTD Phosphatase/Small CTD phosphatase
Haloacid Dehalogenase like Hyrdolase
Unclassified
Phosphatase associated (Co factors such as subunits of multi component PPtases)
Non protein phosphatase (Lipid or sugar phosphatases)

CG	Gene	Gene Name	Gene Group	Gene Family	Human ortholog(s)	RNAi line
CG10089			PTP	ClassI/DSP	DUSP15 DUSP22	17991
CG10138	PpD5	Protein phosphatase D5	STP	PPP		104452
CG10371	Plip	PTEN-like phosphatase	PTP	CLASSICAL	PTPMT1	104774
CG10376	PP2C		STP	PPM	PPM1F PPM1E	101335 35473
CG10417			STP	PPM	PPM1G	106180

CG10426			Non Protein Phosphatase	Lipid Phosphatase	INPP5E	16048
CG10443	Lar	Leukocyte-antigen-related-like	Phosphatase	CLASSICAL	PTPRS PTPRD PTPRF	107996
CG10493	dPHLPP /PP2C	H domain leucine-rich repeat protein phosphatase	STP	PPM	PHLPP1, PHLPP2	16057
CG10930	PpY-55A	Protein phosphatase Y at 55A	STP	PPP		102021 16096
CG10975	Ptp69D	Protein tyrosine phosphatase 69D	PTP	CLASSICAL	PTPRG PTPRZ1	104761
CG11217	CanB2	Calcineurin B2	STP	PPP	PPP3R 1PPP3R2 ,WDR92	104370
CG11425			Non Protein Phosphatase	Lipid Phosphatase	PPAP2C PPAP2B PPAP2A	103037
CG11426			Non Protein Phosphatase	Lipid Phosphatase	PPAP2C, PPAP2B, PPAP2A	42599
CG11437			Non Protein Phosphatase	Lipid Phosphatase	PPAP2C, PPAP2B, PPAP2A	9452
CG11438			Non Protein Phosphatase	Lipid Phosphatase	PPAP2C, PPAP2B, PPAP2A	11438 R-3
CG11440	laza		Non Protein Phosphatase	Lipid Phosphatase	PPAP2C, PPAP2B, PPAP2A	42592
CG11516	Ptp99A	Protein tyrosine phosphatase 99A	PTP	CLASSICAL	PTPRG PTPRZ1	103457

CG11597	PP4-like		STP	PPP		104729 38541
CG12078			Asp based catalysis	HAD		101274
CG12091			STP	PPM	PPTC7	100800
CG12151	Pdp	Pyruvate dehydrogenase phosphatase	STP	PPM		107271
CG12169	Ppm1	Ppm1	STP	PPP		101257
CG12217	PpV	Protein phosphatase V	STP	PPP	PPP2CA PPP2CB PPP4C PPP6C	101997
CG12252	Fcp1		Asp based catalysis	FCP/SCP		106253
CG1228	Ptpmeg	ptpmeg	PTP	CLASSICAL	PTPN3, PTPN4	103740
CG12746			Non Protein Phosphatase	Lipid Phosphatase		104989
CG13197			PTP	Class I/DSP	DUSP11	105122
CG13311			Phophastase associated	Phosphatase associated		101655
CG1395	stg	string	PTP	ClassI/DSP	CDC25A CDC25B	17760
CG14211	MKP-4	MAPK Phosphatase 4	PTP	ClassI/DSP	DUSP12	104884
CG14212			?	HAD	PHOSPHO1 PHOSPHO2	104555
CG14216	Ssu71	Ssu72 CTD phosphatase	UNCLASSIFIED SERINE/THREONINE PHOSPHATASES	RNA pol II phosphatase	SSU72	104388
CG14297			PTP	ClassIII		102071
CG14411			PTP	ClassI/DSP	MTMR0 MTMR11 MTMR12	17576

CG1455	CanA1	Calcineurin A1	STP	PPP	PPP3CB PPP3CC	32283
CG15528			PTP	ClassI/DSP	DUSP14, DUSP21	105484
CG15743			Non Protein Phosphatase	Lipid Phosphatase		42685
CG15771			HALOACID DEHALOGENASE NON-PROTEIN PHOSPHATASES	HAD	NANP	106331
CG15912			HALOACID DEHALOGENASE NON-PROTEIN PHOSPHATASES	HAD	HDHD3	100777
CG1696	Dd	Dullard	Asp based catalysis	FCP/SCP	CTDNEP1	104785
CG17026			Non Protein Phosphatase	Lipid Phosphatase	IMPA1 IMPA2	32813
CG17027			Non Protein Phosphatase	Lipid Phosphatase	IMPA1 IMPA2	103270
CG17028			Non Protein Phosphatase	Lipid Phosphatase	IMPA1 IMPA2	100100
CG17029			Non Protein Phosphatase	Lipid Phosphatase	IMPA1 IMPA2	32822
CG17291	Pp2A-29B	Protein phosphatase 2A at 29B	STP	PPM	PPM1H PPM1J PPM1M	49671
CG17598			STP	PPM	PPM1H PPM1J PPM1M	32955
CG17746			STP	PPM		100178
CG1817	Ptp10D	Protein tyrosine phosphatase 10D	PTP	CLASSICAL	PTPRB PTPRJ PTPRO	1101
CG18243	Ptp52F	Ptp52F	PTP	CLASSICAL		3116
CG1906	alph	alphabet	STP	PPM	PPM1A PPM1B	105483 32476
CG2096	flw	flapwing	STP	PPP	PPP1CA PPP1CB	104677

					PPP1CC	
CG2890	PPP4-R2	Protein phosphatase 4 regulatory subunit 2-related	STP	PPP	PPP4R2	25445
CG2984	Pp2C1	Protein phosphatase 2C	STP	PPM	PP2CM	105249
CG3028	Ipp	nositol polyphosphate 1-phosphatase	Non Protein Phosphatase	Lipid Phosphatase	INPP1	25615
CG31692	fbp	fructose-1,6-bisphosphatase	Non Protein Phosphatase	Carbohydrate Phosphatase	FBP1 FBP2	21396
CG31795	ia2	ia2 ortholog	PTP	CLASSICAL	PTPRN PTPRN2	7560
CG3200	Reg-2		HAD	HAD	HDHD3	107185
CG32156	Mbs	Myosin binding subunit	STP	PPP	PP1R12A PP1R12B	105762
CG3245	PpN58A	Protein phosphatase N at 58A	STP	PPP	PP2CA PP2CB PPP4C PPP6C	102060
CG32505	PP4-19C	Protein phosphatase 19C	STP	PPP	PP2CA PP2CB PPP4C PPP6C	25317
CG32697	l(1)G0232		PTP	CLASSICAL	PTPN9	104427
CG32812			STP	PPP		104081
CG33747	primo 2	primo 2	PTP	ClassIII	ACP2	50642
CG34099	Mkp	MAP kinase-specific phosphatase	PTP	ClassI/DSP	DUSP19	104374
CG3530			PTP	ClassI/DSP	MTM6 MTM7 MTM8	26216
CG3573	Ocrl	Oculocerebrorenal syndrome of Lowe ortholog	Non Protein Phosphatase	Lipid Phosphatase	OCRL INPB5B	34649
CG3632			PTP	ClassI/DSP	MTM3 MTM4	3632R-3 (III)
CG3705	aay	astray	Asp based catalysis	HAD	PSPH	23179
CG3954	csw	corkscrew	PTP	CLASSICAL	PTPN6 PTPN11	21756
CG4123	Mipp1	Multiple inositol polyphosphate	Non protein phosphatase	Lipid Phosphatase		101634

		phosphatase 1				
CG4209	CanB	Calcineurin B	STP	PPP	PPP3R1 PPP3R2 WDR92	21611
CG4317	Mipp2	Multiple inositol polyphosphate phosphatase 2	Non Protein Phosphatase	Lipid Phosphatase		108018
CG4993	PRL-1	PRL-1	PTP	ClassI/DSP		107836
CG5026			PTP	ClassI/DSP	MTMR9	105674 34915
CG5565			HAD	HAD	PUDP	100396
CG5643	wdb	widerborst	STP	PPP	PPP2R5A PPP2R5C PPP2R5D PPP2R5E	101406
CG5671	Pten	Pten	PTP	ClassI/DSP	PTEN TPTE TPTE2	101475
CG5830			Asp based catalysis	FCP/PCP	CTDSP1 CTDSP2 CTDSPL	101539
CG6036			STP	PPM	PPM1A PPM1B	105568
CG6235	tws	twins	STP	PPP	PPP2CA PPP2CB PPP4C PPP6C	104167
CG6238	ssh	slingshot	PTP	ClassI/DSP	SSH1 SSH2 SSH3	107998 6238 R-4
CG6562	synj	synaptojanin	Non Protein Phosphatase	Lipid Phosphatase	SYNJ1 SYNJ2	46070
CG6571	rdgC	retinal degeneration C	STP	PPP	PPEF1 PPEF2	35105
CG6593	Ppla 96a	Protein phosphatase 1α at 96A	STP	PPP	PPP1CA PPP1CB PPP1CC	105525

CG6697	Ublcp1	Ubiquitin-like domain-containing C-terminal domain phosphatase 1	HAD	HAD		27719
CG6805			Non Protein Phosphatase	Lipid Phosphatase	INPP5J INPP5K	107728
CG6899	Ptp4E	Protein tyrosine phosphatase 4E	PTP	CLASSICAL		1012
CG7109	MTS/PP2A	microtubule star	STP	PPP	PPP2CA PPP2CB PPP4C PPP6C	35171
CG7115	PP2C		STP	PPP	PPM1L	103354 9404
CG7180			PTP	CLASSICAL	PTPRK	102397
CG7378			PTP	ClassI/DSP	DUSP1 DUSP3 DUSP13 DUSP26 DUSP27	106098
CG7850	puc	puckered	PTP	ClassI/DSP	DUSP10	3018
CG8402	PpD3	Protein phosphatase 3	STP	PPP	PPP5C	107386 24308
CG8584			Asp based catalysis	FCO/SCP		107849
CG8804	wun	wunen	Non Protein Phosphatase	Lipid Phophatase	PPAP2A PPAP2B PPAP2C	6446
CG8805	wun2	wunen	Non Protein Phosphatase	Lipid Phophatase	PPAP2A PPAP2B PPAP2C	103452 4176
CG8822	PpD6	Protein phosphatase 6	STP	PPP	PPP1CA PPP1CB PPP1CC	104211 36047
CG9115	mtm	myotubularin	PTP	ClassI/DSP	MTM1 MTMR1 MTMR2	29032
CG9128	Sac1	Sac1	Non Protein Phosphatase	Lipid Phosphatase	SACM1L	37216

CG9151	acj6	abnormal chemosensory jump 6	ss	Phosphatase associated	CIB1 CIB2 CIB4	105292
CG9156	Pp1-13C	Protein phosphatase at 13C	STP	PPP	PPP1CA PPP1CB PPP1CC	9156-R1 (II)
CG9181	Ptp61F	Protein tyrosine phosphatase 61F	PTP	CLASSICAL	PTPN1 PTPN2	37436
CG9311	mop	myopic	PTP	CLASSICAL	PTPN23	104860
CG9351	flfl	falafel	STP	PPP	SMEK1 SMEK2	24143
CG9389			Non Protein Phosphatase	Lipid Phosphatase	IMPA1	44663
CG9391			Non Protein Phosphatase	Lipid Phosphatase	IMPA1 IMPA2	23723
CG9493	Pez	Pez	PTP	CLASSICAL	PTPN14	40743
CG9554	eya	eyes absent	Asp based catalysis	HAD	EYA1 EYA2 EYA3 EYA4	108071
CG9578	Tip41			Phosphatase Associated	TIPRL	9578-R3 (II) 9578-R4 (III)
CG9784			Non Protein Phosphatase	Lipid Phosphatase		30098
CG9819	CanA-14F	Calicneurin A at 14F	STP	PPP		30105
CG9842	Pp2B-14D	Protein phosphatase 2B at 14D	STP	PPP	PPP3CB PPP3CC	103144 46873
CG9856	PTP-ER	Protein tyrosine phosphatase ERK/Enhancer of Ras1	PTP	ClassI/DSP		9856 R-1

Phosphatases and phosphatase-associated factors assayed in *Drosophila* adult eye cell polarity modifier screens (sorted by CG number) encoding either phosphatases or associated factors for which *UAS*-controlled inverted repeat strains were tested. Phosphatase group and family is indicated (see color-coded chart). PTP and STP refer to Protein Tyrosine or Serine/Threonine Phosphatases respectively, as well as human orthologs. RNAi line refers to VDRC number and those numbers denoted with R were obtained from NIG (see *Drosophila* stocks, Materials and Methods for further information).

Table S3. Gene list of *GMR* > *crb^intra^*, *GMR* > *aPKC^CA^* and *GMR* modifiers

CG Number	VDRC or NIG Number	Gene	*GMR-gal4*	*GMR>crb^intra^*	*GMR>aPKC^CA^*
**CLASS ONE**					
CG9856	9856 R-1	PTP-ER	NE	NE	small, glassy
CG9222	104259		NE	NE	NE (larger?)
CG8657	4659	Dgk	NE	NE	small, glassy
CG8402	107386	PpD3	NE	NE	more necrotic
CG8351	28895	Tcp-1-eta1	NE	NE	small, glassy
CG8049	106962	Btk29A	NE	NE	less necrotic
CG7892	101545	nmo	NE	NE	larger, glassy
CG7643	7643 R-1	ald	NE	[1]NE	smaller, necrotic
CG7497	106421		NE	NE	more necrotic
CG7186	105102	SAK	NE	NE	less necrotic
CG7028	107042	PRP4	NE	NE	smaller
CG6697	27719	Ublcp1	NE	NE	less necrotic

CG6518	2895	inaC	NE	NE	more necrotic
CG6355	27591	fab1	NE	NE	smaller, less necrotic
CG6297	107001	JIL-1	NE	NE	less necrotic
CG6036	105568		NE	NE	less necrotic
CG5680	34138[i]	bsk	NE	NE	more necrotic
CG5483	105630	lrrk	NE	NE	less necrotic
CG5408	106774	trbl	NE	NE	larger?
CG4926	932	Ror	NE	NE	glassy, less necrotic
CG4839	26641	PRKG-like	NE	NE	more necrotic
CG4803	104701	Takl2	NE	NE	small, glassy
CG4209	21611	CanB	NE	NE	less necrotic
CG4141	38985	DP110 - PI3K	NE	NE	enhanced, smaller
CG4141	2902	DP110 - PI3K	NE	NE	enhanced, smaller
CG33554	2905 R-3	Nipped-A	NE	NE	small, glassy
CG33519	106267	Unc-89	NE	NE	smaller, necrotic
CG32743	4549 R-1	Smg1	NE	NE	smaller, strongly necrotic
CG32717	100685	sdt	NE	NE	more necrotic
CG31873	105621	Mulk	NE	NE	less necrotic
CG31349	38863	pyd	NE	NE	less necrotic

CG2845	20909	D-Raf - Phl	NE	NE	enhanced, smaller
CG2577	41693	CKIa-like	NE	NE	undulated @18ºC
CG2096	104677	flw	NE	NE	small, glassy, necrotic
CG1696	104785	Dd	NE	NE	more necrotic
CG13197	105122		NE	NE	less necrotic
CG12559	43123	rl	NE	NE	small, glassy
CG11870	16334	Emk	NE	NE	glassy, less necrotic
CG11621	16239	Pi3K68D	NE	NE	slightly larger
CG10930	102021	PpY-55A	NE	NE	small, necrotic
CG10702	100842		NE	NE	more necrotic
CG10572	107187	Cdk8	NE	NE	less necrotic
CG10371	104774	Plip	NE	NE	less necrotic
CG10138	104452	PpD5	NE	NE	more necrotic
**CLASS TWO**					
CG9774	3793	Ppm1	NE	glassy, ruffled edge	NE
CG9578	9578-R4	Reg-2	NE	glassy, ruffled edge	NE

CG8914	102633		NE	glassy, ruffled edge	NE
CG8866	103725	p38c	NE	glassy, ruffled edge	NE
CG33338	105173		NE	glassy, ruffled edge	NE
CG32484	101018	Sk2	NE	glassy, ruffled edge	NE
CG3200	107185		NE	glassy, ruffled edge	NE
CG1830	33054	PhKγ	NE	glassy, ruffled edge	NE
CG17216	105265	KP78b	NE	S, less rough	NE
CG15793	107276		NE	glassy, ruffled edge	NE
CG1389	1016	Dsor1	NE	S, narrower	NE
CG13850	100863	CKIa-like	NE	bulgy, glassy edge	NE
CG12169	101257	CKIa-like	NE	undulated posterior	NE
CG12147	101875		NE	Fem – mildly ruffled edge, males - NE	NE
CG12147	31658		NE	mildly rougher	NE
CG12091	100800	Tip41	NE	roughness, few necrotic ommatidia	NE
CG11489	102632		NE	bulgy, glassy edge, smaller?	NE
CG10417	106180		NE	slightly overgrown, folded	NE
CG10082	103749		NE	mildly smaller / glassier	NE

CLASS THREE

CG9784	30098		NE	folded, necrotic	smaller, necrotic
CG8967	104688	otk	NE	glassy, ruffled edge	more necrotic
CG8878	100985		NE	S, narrower	small, glassy, necrotic
CG8878	28970		NE	glassier edges, mildly depigmented	smaller, more necrotic / rescued @18°C
CG8874	107266	Fps85D	NE	less rough	small, necrotic
CG8805	4176	wun2	NE	NE	small, glassy, necrotic.
CG8805	103452	wun2	NE	glassy, ruffled edge	NE
CG8485	35939	SAP/SNRK	NE	slightly glassy around edges	smaller
CG8224	853	babo	NE	narrower	smaller
CG7693	106919	fray	NE	less rough	small, necrotic
CG7597	25508		NE	folded, necrotic	more necrotic, semi lethal
CG7378	106098		NE	bulgy, glassy edge	more necrotic
CG7125	106255	PKD	NE	S, less rough	small, glassy
CG7115	103354		NE	glassy, ruffled edge	more necrotic
CG7097	103580	hppy	NE	glassy, ruffled edge	more necrotic, larger
CG7097	35166	hppy	NE	slightly glassy edges	enhanced, stronger /mildly glassier @18°C
CG6963	106826	gish	NE	bulgy, glassy edge	less necrotic

CG6963	26003	gish	NE	NE	glassier
CG6703	34184	CASK	NE	mildly undulated posterior	undulated @18°C
CG6703	104793	CASK	NE	glassy, ruffled edge	NE
CG6620	104051	ial	NE	S, narrower	small, glassy
CG6238	6238 R-4	ssh	NE	depigmented, larger	small, glassy
CG6114	100717		NE	glassy, ruffled edge	more necrotic
CG5974	103774	pll	NE	bulgy, glassy, ruffled edge	pupal lethal
CG5725	101437	fbl	NE	glassy, ruffled edge	more necrotic
CG5671	101475	Pten	NE	glassy, ruffled edge, necrotic	more necrotic, larger (?)
CG5643	101406	wdb	NE	glassy, larger	more necrotic
CG5565	100396		NE	glassy, ruffled edge	more necrotic
CG5387	34990	Cdk5	NE	small, glassy	smaller
CG5373	100296	Pi3K59F	NE	glassy, ruffled edge	more necrotic
CG5182	101146	Pk34A	NE	glassy edge, smaller	small, glassy, necrotic
CG5182	27368	Pk34A	NE	NE	more necrotic
CG5179	103561	Cdk9	NE	lethal	small, necrotic, semi lethal
CG5179	30448	Cdk9	NE	pupal lethal	pupal lethal
CG4379	101524	Pka-C1	NE	glassy, ruffled edge, necrotic	more necrotic, smaller

CG4268	107303	Pitslre	NE	glassy, ruffled edge	more necrotic
CG3915	102192	Drl-2	NE	ruffled edge, mildly bulgy	more necrotic
CG3837	105549		NE	glassy, ruffled edge	Larger, less necrotic
CG34384	102481		NE	glassy, ruffled edge	more necrotic
CG34357	105185		NE	less rough	more necrotic
CG31127	1214	Wsck	NE	folded, overgrown	more necrotic
CG30021	29965	skf	NE	less rough	glassy, less necrotic
CG2890	25445	PPP4-R2	NE	glassy	glassier, but not necrotic
CG2048	9241	dco	NE	ruffled edge	small, glassy
CG1906	105483	alph	NE	glassy, ruffled edge	small, necrotic
CG18604	20655		NE	ruffled edge & eye	more necrotic / glassy @18°C
CG18582	46043	mbt	NE	smaller, glassy edge	smaller, glassy
CG18492	101357	Tak1	NE	less rough	Larger
CG18492	1388R-1	Tak1	NE	few necrotic ommatidia	almost normal @18°C
CG17698	105884		NE	bulgy, glassy edge	more necrotic, larger
CG17348	100039	drl	NE	glassy, ruffled edge	more necrotic
CG17256	103408	Nek2	NE	glassy, ruffled edge	more necrotic
CG17090	32854	HIPK	NE	smaller, glassier at posterior edge	smaller, glassy @ 18°C and 25°C

CG16910	100257	key	NE	glassy, ruffled edge	more necrotic
CG1594	102830	hop	NE	glassy, ruffled edge	more necrotic
CG15224	106845	CkII	NE	glassy, ruffled edge, necrotic	more necrotic, smaller
CG15072	39864	SIK3	NE	undulated posterior	small, glassy @18°C
CG14895	107260	Pak3	NE	glassy, ruffled edge, necrotic	more necrotic, smaller
CG14217	17432	Tao-1	NE	undulated posterior	more necrotic / undulated @18°C
CG14212	104555		NE	glassy, ruffled edge	small, glassy, necrotic
CG14026	105834	tkv	NE	small, glassy	pupal pharate lethal
CG12306	20177	POLO	NE	glassy posterior	smaller, glassy @18°C
CG12244	106822	lic	NE	glassy, ruffled edge	more necrotic
CG11516	103457	Ptp99A	NE	glassy, ruffled edge, necrotic	larger, glassy, mildly necrotic
CG11217	104370	CanB2	NE	glassy, ruffled edge	more necrotic / larger?
CG1107	103426	aux	NE	necrotic, folded	small, necrotic
CG1107	16182	aux	NE	small, glassy	smaller
CG10975	104761	Ptp69D	NE	glassy, ruffled edge	less necrotic
CG10522	10522 R-1	sti	NE	narrower	small, glassy, bit necrotic
CG10443	107996	Lar	NE	glassy, ruffled edge	larger, necrotic
CG10295	12553	PAK1	NE	bulgy posterior	enhanced, smaller

CG10261	105624	aPKC	NE	less rough	near perfect rescue
CG10261	2907	aPKC	NE	S, mild suppression	strong suppression
CG10260	105614		NE	glassy, ruffled edge	more necrotic
CG7850	3018	puc	NE	narrower, semi lethal	eye ablated, semi lethal
CG4290	103739	SIK2	NE	narrower, glassy	small, glassy
CG2984	105249	Pp2C1	NE	glassy, ruffled edge	smaller, more necrotic
CG11660	105395		NE	small, glassy	small, glassy
CG1227	105610		NE	small, glassy	pupal lethal
CG14903	105752		NE	glassy, ruffled edge	more necrotic
CG10579	106824	Eip63E	NE	smaller, glassy edge	small, glassy
CG8637	107923	trc	NE	glassy, ruffled edge	small, glassy
CG8637	35988	trc	NE	mildly rougher	NE
**CLASS FOUR**					
CG9985	101624	sktl	males only – necrotic patches	narrower, glassy	small, glassy, semi lethal
CG9842	46873	PP2B, Ppl2-14D	mildly rough	NE	NE
CG9554	108071	eya	glassy	smaller, glassy, yellow	smaller, strongly necrotic
CG9493	40743	Pez	smaller, rough	small, glassy	pupal lethal, no eyes

CG9351	24143	Flfl (PP4 subunit)	glassy	glassier	Smaller, bulgy, glassy
CG9311	104860		mildly glassy	glassy, ruffled edge	more necrotic
CG9156	9156-R1	PP1-13C	rough, necrotic, semi-lethal	pupal lethal	pupal lethal
CG8822	36047	PP1-like, PpD6	mildly rough	rougher	semi lethal, rougher
CG8222	105353	PP1-like, PpD6	smaller / mildly glassy	small, glassy	small, glassy, semi lethal
CG8222	976	PP1-like, PpD6	necrotic	folded, necrotic	more necrotic
*CG7904	107071	punt	smaller, glassy edge	smaller, glassy edge	pupal lethal
*CG7904	37279	punt	NE	glassy, narrower	smaller, glassier
CG7873	100708	Src42A	smaller, rough	less rough	NE
CG7177	106928		smaller	narrower	small, glassy
CG7004	27785	fwd	bulgy, glassy	bulgy, glassy	pharate lethal
CG6593	105525	Ppl	pharate lethal (eye open)	pupal lethal	pupal lethal
CG6292	103387	CycT	pharate lethal, no eye	pharate lethal	pharate lethal
CG5363	106130	cdc2	mildly narrower & rough	narrower	small, glassy
CG5363	41838	cdc2	mildly rough	less rough	less necrotic
CG5169	107158	GckIII	smaller, mildly glassy	glassy	glassy, less necrotic
CG4527	43783	Slik	slightly depigmented	glassy, slightly bulgy	enhanced, smaller, more necrotic
CG4317	108018	Mipp2	slightly depigmented & bulgy	glassy, ruffled edge	NE
CG34412	105732	tlk	glassy	glassy	smaller, more necrotic

CG3324	34594	Pkg21D	smaller, rough	smaller, rough	pupal lethal
CG32697	104427	l(1)G0232	rough, slightly bulgy	glassy, ruffled edge	small, glassy
CG32505	25317	PP4-19C	glassy	glassy, blistered	rougher, necrotic, not glassy
CG32156	105762	Mbs	rougher, larger	glassy, larger	glassy, necrotic
*CG2615	2615R-1	IKK-eR-1	NE	mildly rough	slightly smaller
*CG2615	2615R-2	IKK-eR-2	mildly rough	mildly rough, bulgy, black spots	slightly smaller
CG2252	51227	fs(1)h	glassy, depigmented edge	pharate lethal	small, necrotic
CG2049	2055R-1	PRK2 (PKN-related	glassy	reduced eye, semi-pupal-lethal	reduced eye, pupal-lethal
CG2028	13664	CkI	smaller / glaced	narrower	small, glassy, semi lethal
CG1891	42457	Sax	mildly rough	rougher	less necrotic
CG18402	991	InR	normal but smaller	less rough	small, glassy
CG17746	100178		mildly rough, bulgy	glassy, bulgy	more glassy
CG17603	106119	Taf1	depigmented, slightly rough	bulgy, larger, scars	larger, less necrotic
CG17342	32885	MNK/LK6	mildly rough	glassy, mildly undulated	stronger rough
CG17291	49671	Pp2A-29B	mildly glassy	ruffled edge	more necrotic, semi lethal
*CG1725	41134	dlg1	almost no eye	almost no eye	pupal lethal, no eyes
*CG1725	41136	dlg1	NE	overgrown, necrotic	more necrotic, semi lethal
CG16973	16973R-1	msn	glassy, rough	smaller, undulated	smaller, undulated
CG1609	103976	Gcn2	smaller, glassy	narrower, glassy	small, glassy

CG14992	39857	Ack	smaller, rougher	small, glassy	smaller, glassy
CG1455	32283	CanA1	pharate lethal	pharate lethal	pharate lethal
CG14211	104884	MKP-4	mildly rough	smaller, glassy edge	pupal lethal
CG1395	17760	stg	mildly rough	less rough	small, glassy
CG12217	101997	PpV	glassy posterior, triangular	glassy, ruffled edge	small, glassy
CG1210	18736	Pk61C	normal but smaller	smaller	small, glassy
CG12072	106174	wts	strongly overgrown	overgrown, semi lethal	larger, more necrotic
CG11597	38541	PP4-like	normal pattern, but no bristles	same roughness, but no bristles	glassier / mild rescue, no bristles @ 18°C
CG11486	106497		smaller, glassy	smaller, glassy edge	pupal lethal
CG11228	104169	hpo	rough, mildly larger	mildly bulgy	larger, more necrotic
*CG11221	100163	PKN	NE	glassy, ruffled edge	NE
*CG11221	42947	PKN	mildly rough, depigmented	mildly folded & overgrown	more necrotic@25°C; folded, necrotic@18°C
CG10539	104369	S6K	slightly smaller	smaller	smaller
CG10498	104959	cdc2c	smaller, glassy	small, glassy	smaller, glassy
CG10079	107130	Egfr	pharate lethal, no eye left	pupal lethal	pupal lethal
CG10033	38319	DG2, for	glassy	glassy, narrower	semi lethal@25°C; reduced, narrow eyes @18°C

---

i * NIG numbers end in R, NE no effect
* Modifier genes classified based on strongest RNAi line phenotype

Results and brief description of adult female eye phenotypes observed for the 185 genes that modified *GMR > crb*^{intra} and/or *GMR > aPKC*^{CA}. A variety of *GMR > crb*^{intra} and/or *GMR > aPKC*^{CA} modified phenotypes were observed including; increased or decreased necrosis, increased growth scored as ‘bulginess’ or ‘folding’, decreased growth described as ‘narrowness’ or small and depigmented or glassy eyes. Gene lists organized by Class. Genes ordered in accordance to CG number, highest to lowest. All interactions are at 25°C unless stated otherwise.

Table S4 Gene list of *en* > *lgli* modifiers

RNAi line	CG	Gene	*en > lgli*	*en > GFP*
	**CLASS 1**			
	**Lethal**			
101624 6229	CG9985	sktl	lethal	lethal
40743	CG9493	Pez	lethal	decreased wing size
104860	CG9311		lethal	lethal
9156-R1 (II)	CG9156	PP1-13C	lethal	lethal
107923 35988	CG8637	trc	lethal	lethal
35939	CG8485	SAP/SNRK	lethal	lethal
28895	CG8351	Tcp-1-etal	lethal	lethal
107071 37279	CG7904	put	lethal	lethal
100708	CG7873	Src42A	lethal	lethal
3018	CG7850	puc	lethal, decreased wing size@RT	lethal
25508	CG7597		lethal	lethal
107042 27808	CG7028		lethal	lethal
27785	CG7004	fwd	lethal	lethal
104051	CG6620	ial	lethal	lethal

105525	CG6593	Ppl	lethal	lethal
101719 2895	CG6518	inaC	lethal, decreased wing size@RT	NE
107998 6238 R4	CG6238	ssh	lethal	decreased wing size
101475	CG5671	Pten	lethal	lethal
34990	CG5387	Cdk5	lethal	lethal
106130 41838	CG5363	cdc2	crumpled, decreased wing size@RT	lethal, decreased wing size@RT
103561 30448	CG5179	Cdk9	lethal	lethal
43783	CG4527	slik	lethal, decreased wing size @RT	NE
105732 46425	CG34412	tlk	lethal	decreased wing size
41693	CG2577	Ckla-like	lethal, crumpled wing@RT	lethal, crumpled wing@RT
51227	CG2252	fs(h)	lethal	lethal
2055R-1	CG2049	PRK2	lethal	lethal
13664	CG2028	CkI	lethal	lethal
101357 1388R-1	CG18492	Tak1	lethal	lethal
991	CG18402	InR	lethal, decreased wing size@RT	lethal, decreased wing size@RT
41134 41136	CG1725	dlg1	lethal	lethal
43783	CG16973	msn	lethal	lethal
39857	CG14992	Ack	lethal	lethal
105834 862	CG14026	tkv	lethal	lethal

106174	CG12072	wts	lethal	lethal
105395	CG11660		lethal	lethal
106497	CG11486		lethal	crumpled wing
104169	CG11228	hpo	lethal, increased wing size @ RT	lethal, increased wing size @ RT
100163 42947	CG11221	PKN	lethal	lethal
103426 16182	CG1107	aux	lethal	lethal
105624 2907	CG10261	aPKC	lethal	lethal
38319	CG10033	DG2, for	lethal	lethal
	**CLASS 2**			
	**Crumpled wing**			
105353 976	CG8222	Pvr	crumpled wing	
107158 49558	CG5169	Gckiii	crumpled wing	crumpled wing
25317	CG32505	PP4-19C	crumpled wing	lethal
104677	CG2096	flw	crumpled, decreased wing size@RT	decreased wing size
46043	CG18582	mbt	crumpled wing	crumpled wing
103976	CG1609	Gcn2	crumpled wing	decreased wing size
43123	CG12559	rl	crumpled wing	crumpled wing

20177	CG12306	20177	crumpled wing	lethal
105610 38647	CG1227		crumpled, decreased wing size@RT	crumpled wing
105614	CG10260		crumpled wing	crumpled wing
	**CLASS 3**			
	**Wing Size**			
30098	CG9784		decreased wing size	NE
100985 28970	CG8878	VRK	decreased wing size@RT	decreased wing size@RT
103725	CG8866		decreased wing size	decreased wing size
103452 4176	CG8805	wun2	decreased wing size	decreased wing size
106962	CG8049	Btk29A	decreased wing size	NE
101545 3002	CG7892	nmo	decreased wing size	decreased wing size
7643 R-1	CG7643	Mps1	decreased wing size	decreased wing size
106098	CG7378		decreased wing size	decreased wing size
106928	CG7177	Wnk	decreased wing size	decreased wing size
103354 9404	CG7115	PP2C	decreased wing size	NE
103580 35166	CG7097	hppy	decreased wing size	decreased wing size
27591	CG6355	fab	decreased wing size	decreased wing size

103774	CG5974	pll	decreased wing size	decreased wing size
101406	CG5643	wdb	decreased wing size@RT	decreased wing size@RT
100396	CG5565		decreased wing size	decreased wing size
932	CG4926	Ror	decreased wing size	NE
101524	CG4379	Pka-C1	decreased wing size	decreased wing size
103739 26496	CG4290	SIK SIK2	decreased wing size	decreased wing size
105185	CG34357		decreased wing size	decreased wing size
102192	CG3915	Drl-2	decreased wing size	decreased wing size
105549	CG3837	Sdr	decreased wing size	decreased wing size
101018	CG32484	Sk2	decreased wing size	decreased wing size
105249	CG2984	Pp2c1	decreased wing size	decreased wing size
9241	CG2048	dco	decreased wing size	NE
105483 32476	CG1906	alph	decreased wing size	decreased wing size
33054	CG1830	Phk	decreased wing size@RT	decreased wing size@RT
105884 35632	CG17698	CAMKIIB	decreased wing size	NE
103408	CG17256	Nek2	decreased wing size	decreased wing size
32854	CG17090	HIPK	decreased wing size	decreased wing size
102830	CG1594	hop	decreased wing size	decreased wing size
39864	CG15072	SIK3	increased wing size	NE

105752	CG14903		decreased wing size	decreased wing size
101997	CG12217	PpV	decreased wing size	decreased wing size
17432	CG14217	Tao-1	increased wing size	increased wing size
104555	CG14212		decreased wing size	decreased wing size
106822	CG12244		decreased wing size	decreased wing size
106088 16334	CG11870	NUAK	decreased wing size	decreased wing size
102021 16096	CG10930	PpY-55A	decreased wing size	decreased wing size
106824	CG10579	Eip63E	decreased wing size	NE
107187 31264	CG10572	Cdk8	decreased wing size	decreased wing size
106180	CG10417		decreased wing size	decreased wing size
101146 27368	CG5182		decreased wing size	decreased wing size

Results and brief description of adult female wing phenotypes observed for the 93 genes that modified *en* > *lgli*. Genes highlighted in blue or yellow were identified as kinases or phosphatase regulators of Wnt signalling respectively (Swarup et al., 2015). Gene lists organized by Class. Genes ordered in accordance to CG number, highest to lowest. RNAi line refers to VDRC number and those numbers denoted with R were obtained from NIG (see *Drosophila* stocks, Materials and Methods for further information). All interactions are at 25°C unless stated otherwise.

Table S5. Gene lists of overlapping modifier genes from Classes 1 - 4 (adult eye cell polarity screen) and *en > lgli* wing screen

Modifiers only in *GMR > aPKC*^{CA} (Class 1 eye screen)

RNAi line	CG	Gene
9856 R-1	CG9856	PTP-ER
104259	CG9222	
4659	CG8657	Dgk
107386	CG8402	PpD3
106421	CG7497	
105102	CG7186	SAK
27719	CG6697	Ublcpl
107001	CG6297	JIL-1
105568	CG6036	
34138	CG5680	bsk
105630	CG5483	IRRK
106774	CG5408	trbl
26641	CG4839	PRKG-like
104701	CG4803	Takl2
21611	CG4209	CanB
38985	CG4141	DP110 - PI3K
2902		Nipped-A
2905 R-3	CG33554	Unc-89
106267	CG33519	Smgl
4549 R-1	CG32743	sdt
100685	CG32717	Mulk
105621	CG31873	pyd
38863	CG31349	D-Raf - Phl
20909	CG2845	Dd
104785	CG1696	
105122	CG13197	Pi3K68D
16239	CG11621	
100842	CG10702	Plip
104774	CG10371	PpD5
104452	CG10138	

Modifiers only in *GMR > crb*^{intra} (Class 2 eye screen)

RNAi line	CG	Gene
3793	CG9774	Ppml
9578-R4	CG9578	Reg-2
102633	CG8914	
105173	CG33338	
107185	CG3200	
105265	CG17216	KP78b
107276	CG15793	Dsorl

1016	CG1389	
100863	CG13850	CKIa-like
101257	CG12169	CKIa-like
101875 31658	CG12147	
100800		
102632	CG12091	Tip41
103749	CG11489	
	CG10082	

Modifiers only in *GMR* > *aPKC*^{CA} and *GMR* > *crb*^{intra} (Class 3 eye screen)

RNAi line	CG	Gene
104688	CG8967	otk
107266	CG8874	Fps85D
853	CG8224	babo
106919	CG7693	fray
106255	CG7125	PKD
106826	CG6963	gish
26003		
104793	CG6703	CASK
34184		
100717	CG6114	
101437	CG5725	fbl
100296	CG5373	Pi3K59F
107303	CG4268	Pitslre
102481	CG34384	Wsck
1214	CG31127	skf
29965	CG30021	PPP4-R2
25445	CG2890	
20655	CG18604	drl
100039	CG17348	key
100257	CG16910	CkII
106845	CG15224	Pak3
107260	CG14895	Ptp99A
103457	CG11516	CanB2
104370	CG11217	Ptp69D
104761	CG10975	sti
10522 R-1	CG10522	Lar
107996	CG10443	PAK1
12553	CG10295	

Modifiers only in *GMR-GAL4* and *GMR* > *aPKC*^{CA} and/or *GMR crb*^{intra} (Class 4 eye screen)

RNAi line	CG	Gene
46873	CG9842	PP2B
108071	CG9554	eya

24143	CG9351	Flfl
104211	CG8822	PP1-like, PpD6
36047		
103387	CG6292	CycT
108018	CG4317	Mipp2
34594	CG3324	Pkg21D
104427	CG32697	l(1)G0232
105762	CG32156	Mbs
2615R-1	CG2615	IKK-eR-1
2615R-2		
42457	CG1891	Sax
100178	CG17746	
106119	CG17603	Tafl
32885	CG17342	MNK/LK6
49671	CG17291	Pp2A-29B
32283	CG1455	CanA1
104884	CG14211	MKP-4
17760	CG1395	stg
18736	CG1210	Pk61C
38541	CG11597	PP4-like
104369	CG10539	S6K
104959	CG10498	cdc2c
107130	CG10079	Egfr

Common modifiers in *en* > *lgli* (or *en-GAL4*) and *GMR* > *aPKC*^{CA} (wing screen and Class 1)

RNAi line	CG	Gene
28895	CG8351	Tcp-1-eta1
107042	CG7028	PRP4
2895	CG6518	inaC
41693	CG2577	CKIa-like
104677	CG2096	flw
43123	CG12559	rl
106962	CG8049	Btk29A
101545	CG7892	nmo
7643 R-1	CG7643	ald
27591	CG6355	fab1
932	CG4926	Ror
16334	CG11870	Emk
102021	CG10930	PpY-55A
107187	CG10572	Cdk8

Common modifiers in *en > lgli* (or *en-GAL4*) and *GMR > crb*^{intra} (wing screen and Class 2)

RNAi line	CG	Gene
103725	CG8866	
101018	CG32484	Sk2
33054	CG1830	PhKγ
106180	CG10417	

Common modifiers in *en > lgli* (or *en-GAL4*), *GMR > aPKC*^{CA} and *GMR > crb*^{intra} (wing screen and Class 3)

RNAi line	CG	Gene
107923 35988	CG8637	trc
35939	CG8485	SAP/SNRK
3018	CG7850	puc
25508	CG7597	
104051	CG6620	ial
107998 6238 R4	CG6238	ssh
101475		
	CG5671	Pten
34990		
	CG5387	Cdk5
103561 30448		
	CG5179	Cdk9
101357 1388R-1		
105834 862	CG18492	Tak1
105395		
	CG14026	tkv
103426 16182		
105624 2907		
	CG11660	
46043	CG1107	aux
20177		
	CG10261	aPKC
105610 38647		
105614		
	CG18582	mbt
30098		
	CG12306	POLO
100985 28970	CG1227	CG1227
103452 4176		
106098	CG10260	
103354 9404	CG9784	
103580 35166	CG8878	VRK
103774		
	CG8805	wun2
101406		
100396	CG7378	
101524	CG7115	PP2C
103739 26496		
	CG7097	hppy
105185		
102192		
	CG5974	pll
105549		
	CG5643	wdb
105249		
	CG5565	

9241	CG4379	Pka-C1
105483 32476	CG4290	SIK SIK2
105884 35632	CG34357	
103408	CG3915	Drl-2
32854	CG3837	Sdr
102830	CG2984	Pp2c1
39864	CG2048	dco
105752	CG1906	alph
17432	CG17698	CAMKIIB
104555	CG17256	Nek2
106822	CG17090	HIPK
106824	CG1594	hop
101146 27368	CG15072	SIK3
	CG14903	
	CG14217	Tao-1
	CG14212	
	CG12244	
	CG10579	Eip63E
	CG5182	

Common modifiers in *en > lgli* (or *en-GAL4*) and *GMR-GAL4* and *GMR > aPKC*^{CA} and/or *GMR > crb*^{intra} (Class 4).

RNAi line	CG	Gene
101624 6229	CG9985	sktl
40743	CG9493	Pez
104860	CG9311	
9156-R1 (II)	CG9156	PP1-13C
107071 37279	CG7904	put
100708	CG7873	Src42A
27785	CG7004	fwd
105525	CG6593	Ppl
106130 41838	CG5363	cdc2
43783	CG4527	slik
105732 46425	CG34412	tlk
51227	CG2252	fs(h)
2055R-1	CG2049	PRK2
13664	CG2028	CkI
991	CG18402	InR
41134 41136	CG1725	dlg
16973R-1 (III)	CG16973	msn
39857	CG14992	Ack

106174	CG12072	wts
106497	CG11486	
104169	CG11228	hpo
100163 42947<br>38319	CG11221<br>CG10033	PKN<br>DG2, for
105353 976	CG8222	Pvr
107158 49558	CG5169	Gckiii
25317	CG32505	PP4-19C
103976	CG1609	Gcn2
106928	CG7177	Wnk
101997	CG12217	PpV

Gene lists organized by Class. Genes ordered in accordance to CG number, highest to lowest. RNAi line refers to VDRC number and those numbers denoted with R were obtained from NIG (see *Drosophila* stocks, Materials and Methods for further information).

Table S6. *en* > *lgli* Group 3 Wing Size Subclasses

Group 3 Wing size Subclasses	Gene
Subclass 3.1: *en > lgli* only modifier	*Btk29A* , *PP2C* , *CG9784, Ror, dco, CAMKIIB, Eip63E*
Subclass 3.2: *en-GAL4* and *en > lgli* modified equivalently	*fabl, Phkγ, nmo, CdK8, ald, NUAK, hppy, Pk34A, alph, *Sk2, *hop, *pll, *lic, * CG8866, *CG14212, *CG14903, *CG7378, *Wnk, *CG10417, *Drl-2, *Nek2, *Sdr, *CG5565, *CG34357*
Subclass 3.3: *en > lgli* smaller than *en-GAL4* alone	*VRK, wdb, Pka-C1, PpV, PpY-55A, SIK2, Pp2cl, wun2, HIPK*
Subclass 3.4: increase in wing size *en > lgli* larger than *en-GAL4* alone	*SIK3, Tao-1*

Analysis of modifier genes that altered *en* > *lgli* wing size compared with *en* – *GAL4* revealed different subclasses. Subclass 1 modifier genes only affected *en* > *lgli* wing size. Subclass 2 modifier genes decreased wing size in both *en* - *GAL4* and *en* > *lgli* equivalently. Subclass 3 modifier genes that impacted *en* > *lgli* wing size to a greater extent than control *en* - *GAL4* wings. Subclass 4 modifier genes increased wing size. Gene lists organized by Class. Genes highlighted in blue or yellow were identified as kinases or phosphatase regulators of Wnt signalling, respectively (Swarup et al., 2015). * Indicate genes identified by KK lines, which may be false positive due to the modification of *tio* gene expression, although these were not interactors with *GMR>crb*. Ones highlighted in red text indicate genes identified by KK lines that also interact with *GMR>crb*, and are potential false positives due to their potential effect on *tio* gene expression.

Table S7. Lists of modifier genes of Wing Size Class 3 that modified *en > lgli*, greater than *en-GAL4* alone (Classes 3.1, 3.3, 3.4).

***en > lgli* Interactor**	***GMR > aPKC***	***GMR > Crb***	***GMR > GAL4***	**Gene Function in signalling pathways**
**Class 3.1**				
Btk29A	+			Src
PP2C	+	+		Wingless
CG9784 (*IPP*) (Inositol phosphate phosphatase)	+	+		Lipid/PI3K
Ror (Receptor Tyrosine Kinase)	+			-
dco	+	+		Wingless, Hedgehog, Hippo, Ras
CAMKIIB (CG17698*)*	+	+		-
Eip63E (Cyclin-dependent protein kinase)	+	+		Wingless, Ras
**Class 3.3**				
VRK (*CG8878*)	+	+		Wingless, Ras
wdb *(PP2A)*	+	+		Wingless, P13K
Pka-C1	+	+		Wingless, Ras, Hedgehog, Dpp
PpV	+	+	+	Wingless, Ras
PpY-55A	+			-
SIK2	+	+		Hippo
Pp2c1	+	+		Wingless
wun2 (Lipid phosphate phosphatase)	+	+		Wingless, Lipid/PI3K
HIPK	+	+		Wingless, Notch, Hippo, Hedgehog, JNK
**Class 3.4**				
SIK3	+	+		Hippo
Tao-1	+	+		Hippo

Gene lists organized by Class. Genes highlighted in blue or yellow were identified as kinases or phosphatase regulators of Wnt signalling respectively (Swarup et al., 2015). Functions of genes in signalling pathways are listed, as revealed from Flybase records.

SUPPLEMENTAL LITERATURE CITED

Swarup, S., T. Pradhan-Sundd, and E.M. Verheyen. 2015. Genome-wide identification of phospho-regulators of Wnt signaling in Drosophila. *Development*. 142:1502-1515.

Tyler, D.M., and N.E. Baker. 2007. Expanded and fat regulate growth and differentiation in the Drosophila eye through multiple signaling pathways. *Dev Biol*. 305:187-201.
