# Supplementary material for: A Kinome RNAi Screen in Drosophila Identifies Novel Genes Interacting with Lgl, aPKC, and Crb Cell Polarity Genes in Epithelial Tissues
Source: G3 (Bethesda). 2017 Jun 13;7(8):2497–509. doi: 10.1534/g3.117.043513 (PMC5555457; doi:10.1534/g3.117.043513)
Supplement: Supplementary file 2 [file 2497TableS5.docx]

**Table S5** Intersection of modifier genes between Classes 1-4 from the adult eye cell polarity and *en > lgli* (or *en-GAL4*) wing screen Class 3 wing size.

Modifiers only in *GMR > aPKC^CA^* (Class 1 eye screen)

| RNAi line | CG | Gene |
| --- | --- | --- |
| \| 9856 R-1 \| \| --- \| \| 104259 \| \| 4659 \| \| 107386 \| \| 106421 \| \| 105102 \| \| 27719 \| \| 107001 \| \| 105568 \| \| 34138 \| \| 105630 \| \| 106774 \| \| 26641 \| \| 104701 \| \| 21611 \| \| 38985  2902 \| \| 2905 R-3 \| \| 106267 \| \| 4549 R-1 \| \| 100685 \| \| 105621 \| \| 38863 \| \| 20909 \| \| 104785 \| \| 105122 \| \| 16239 \| \| 100842 \| \| 104774 \| \| 104452 \| | \| CG9856 \| \| --- \| \| CG9222 \| \| CG8657 \| \| CG8402 \| \| CG7497 \| \| CG7186 \| \| CG6697 \| \| CG6297 \| \| CG6036 \| \| CG5680 \| \| CG5483 \| \| CG5408 \| \| CG4839 \| \| CG4803 \| \| CG4209 \| \| CG4141 \| \| CG33554 \| \| CG33519 \| \| CG32743 \| \| CG32717 \| \| CG31873 \| \| CG31349 \| \| CG2845 \| \| CG1696 \| \| CG13197 \| \| CG11621 \| \| CG10702 \| \| CG10371 \| \| CG10138 \| | \| PTP-ER \| \| --- \| \|  \| \| Dgk \| \| PpD3 \| \| SAK \| \| Ublcp1 \| \| JIL-1 \| \|  \| \| bsk \| \| IRRK \| \| trbl \| \| PRKG-like \| \| Takl2 \| \| CanB \| \| DP110 - PI3K \| \| Nipped-A \| \| Unc-89 \| \| Smg1 \| \| sdt \| \| Mulk \| \| pyd \| \| D-Raf - Phl \| \| Dd \| \|  \| \| Pi3K68D \| \|  \| \| Plip \| \| PpD5 \| |

Modifiers only in *GMR > crb^intra^* (Class 2 eye screen)

| RNAi line | CG | Gene |
| --- | --- | --- |
| \| 3793 \| \| --- \| \| 9578-R4 \| \| 102633 \| \| 105173 \| \| 107185 \| \| 105265 \| \| 107276 \| \| 1016 \| \| 100863 \| \| 101257 \| \| 101875 31658 \| \| 100800 \| \| 102632 \| \| 103749 \| | \| CG9774 \| \| --- \| \| CG9578 \| \| CG8914 \| \| CG33338 \| \| CG3200 \| \| CG17216 \| \| CG15793 \| \| CG1389 \| \| CG13850 \| \| CG12169 \| \| CG12147 \| \| CG12091 \| \| CG11489 \| \| CG10082 \| | \| Ppm1 \| \| --- \| \| Reg-2 \| \|  \| \| KP78b \| \| Dsor1 \| \|  \| \| CKIa-like \| \| CKIa-like \| \|  \| \| Tip41 \| \|  \| \|  \| |

Modifiers only in *GMR > aPKC^CA^* and *GMR > crb^intra^* (Class 3 eye screen)

| RNAi line | CG | Gene |
| --- | --- | --- |
| \| 104688 \| \| --- \| \| 107266 \| \| 853 \| \| 106919 \| \| 106255 \| \| 106826  26003 \| \| 104793  34184 \| \| 100717 \| \| 101437 \| \| 100296 \| \| 107303 \| \| 102481 \| \| 1214 \| \| 29965 \| \| 25445 \| \| 20655 \| \| 100039 \| \| 100257 \| \| 106845 \| \| 107260 \| \| 103457 \| \| 104370 \| \| 104761 \| \| 10522 R-1 \| \| 107996 \| \| 12553 \| | \| CG8967 \| \| --- \| \| CG8874 \| \| CG8224 \| \| CG7693 \| \| CG7125 \| \| CG6963 \| \| CG6703 \| \| CG6114 \| \| CG5725 \| \| CG5373 \| \| CG4268 \| \| CG34384 \| \| CG31127 \| \| CG30021 \| \| CG2890 \| \| CG18604 \| \| CG17348 \| \| CG16910 \| \| CG15224 \| \| CG14895 \| \| CG11516 \| \| CG11217 \| \| CG10975 \| \| CG10522 \| \| CG10443 \| \| CG10295 \| | \| otk \| \| --- \| \| Fps85D \| \| babo \| \| fray \| \| PKD \| \| gish \| \| CASK \| \|  \| \| fbl \| \| Pi3K59F \| \| Pitslre \| \| Wsck \| \| skf \| \| PPP4-R2 \| \|  \| \| drl \| \| key \| \| CkII \| \| Pak3 \| \| Ptp99A \| \| CanB2 \| \| Ptp69D \| \| sti \| \| Lar \| \| PAK1 \| |

Modifiers only in *GMR-GAL4* and *GMR > aPKC^CA^* and/or *GMR crb^intra^* (Class 4 eye screen)

| RNAi line | CG | Gene |
| --- | --- | --- |
| \| 46873 \| \| --- \| \| 108071 \| \| 24143 \| \| 104211  36047 \| \| 103387 \| \| 108018 \| \| 34594 \| \| 104427 \| \| 105762 \| \| 2615R-1  2615R-2 \| \| 42457 \| \| 100178 \| \| 106119 \| \| 32885 \| \| 49671 \| \| 32283 \| \| 104884 \| \| 17760 \| \| 18736 \| \| 38541 \| \| 104369 \| \| 104959 \| \| 107130 \| | \| CG9842 \| \| --- \| \| CG9554 \| \| CG9351 \| \| CG8822 \| \| CG6292 \| \| CG4317 \| \| CG3324 \| \| CG32697 \| \| CG32156 \| \| CG2615 \| \| CG1891 \| \| CG17746 \| \| CG17603 \| \| CG17342 \| \| CG17291 \| \| CG1455 \| \| CG14211 \| \| CG1395 \| \| CG1210 \| \| CG11597 \| \| CG10539 \| \| CG10498 \| \| CG10079 \| | \| PP2B \| \| --- \| \| eya \| \| Flfl \| \| PP1-like, PpD6 \| \| CycT \| \| Mipp2 \| \| Pkg21D \| \| l(1)G0232 \| \| Mbs \| \| IKK-eR-1 \| \| Sax \| \|  \| \| Taf1 \| \| MNK/LK6 \| \| Pp2A-29B \| \| CanA1 \| \| MKP-4 \| \| stg \| \| Pk61C \| \| PP4-like \| \| S6K \| \| cdc2c \| \| Egfr \| |

Common modifiers in *en > lgli* (or *en-GAL4*) and *GMR > aPKC^CA^* (wing screen and Class 1)

| RNAi line | CG | Gene |
| --- | --- | --- |
| \| 28895 \| \| --- \| \| 107042 \| \| 2895 \| \| 41693 \| \| 104677 \| \| 43123 \| \| 106962 \| \| 101545 \| \| 7643 R-1 \| \| 27591 \| \| 932 \| \| 16334 \| \| 102021 \| \| 107187 \| | \| CG8351 \| \| --- \| \| CG7028 \| \| CG6518 \| \| CG2577 \| \| CG2096 \| \| CG12559 \| \| CG8049 \| \| CG7892 \| \| CG7643 \| \| CG6355 \| \| CG4926 \| \| CG11870 \| \| CG10930 \| \| CG10572 \| | \| Tcp-1-eta1 \| \| --- \| \| PRP4 \| \| inaC \| \| CKIa-like \| \| flw \| \| rl \| \| Btk29A \| \| nmo \| \| ald \| \| fab1 \| \| Ror \| \| Emk \| \| PpY-55A \| \| Cdk8 \| |

Common modifiers in *en > lgli* (or *en-GAL4*) and *GMR > crb^intra^* (wing screen and Class 2)

| RNAi line | CG | Gene |
| --- | --- | --- |
| \| 103725 \| \| --- \| \| 101018 \| \| 33054 \| \| 106180 \| | \| CG8866 \| \| --- \| \| CG32484 \| \| CG1830 \| \| CG10417 \| | \|  \| \| --- \| \| Sk2 \| \| PhKγ \| \|  \| |

Common modifiers in *en > lgli* (or *en-GAL4*), *GMR > aPKC^CA^* and *GMR > crb^intra^* (wing screen and Class 3)

| RNAi line | CG | Gene |
| --- | --- | --- |
| \| 107923 35988 \| \| --- \| \| 35939 \| \| 3018 \| \| 25508 \| \| 104051 \| \| 107998 6238 R4 \| \| 101475 \| \| 34990 \| \| 103561 30448 \| \| 101357 1388R-1 \| \| 105834 862 \| \| 105395 \| \| 103426 16182 \| \| 105624 2907 \| \| 46043 \| \| 20177 \| \| 105610 38647 \| \| 105614 \| \| 30098 \| \| 100985 28970 \| \| 103452 4176 \| \| 106098 \| \| 103354 9404 \| \| 103580 35166 \| \| 103774 \| \| 101406 \| \| 100396 \| \| 101524 \| \| 103739 26496 \| \| 105185 \| \| 102192 \| \| 105549 \| \| 105249 \| \| 9241 \| \| 105483 32476 \| \| 105884 35632 \| \| 103408 \| \| 32854 \| \| 102830 \| \| 39864 \| \| 105752 \| \| 17432 \| \| 104555 \| \| 106822 \| \| 106824 \| \| 101146 27368 \| | \| CG8637 \| \| --- \| \| CG8485 \| \| CG7850 \| \| CG7597 \| \| CG6620 \| \| CG6238 \| \| CG5671 \| \| CG5387 \| \| CG5179 \| \| CG18492 \| \| CG14026 \| \| CG11660 \| \| CG1107 \| \| CG10261 \| \| CG18582 \| \| CG12306 \| \| CG1227 \| \| CG10260 \| \| CG9784 \| \| CG8878 \| \| CG8805 \| \| CG7378 \| \| CG7115 \| \| CG7097 \| \| CG5974 \| \| CG5643 \| \| CG5565 \| \| CG4379 \| \| CG4290 \| \| CG34357 \| \| CG3915 \| \| CG3837 \| \| CG2984 \| \| CG2048 \| \| CG1906 \| \| CG17698 \| \| CG17256 \| \| CG17090 \| \| CG1594 \| \| CG15072 \| \| CG14903 \| \| CG14217 \| \| CG14212 \| \| CG12244 \| \| CG10579 \| \| CG5182 \| | \| trc \| \| --- \| \| SAP/SNRK \| \| puc \| \|  \| \| ial \| \| ssh \| \| Pten \| \| Cdk5 \| \| Cdk9 \| \| Tak1 \| \| tkv \| \|  \| \| aux \| \| aPKC \| \| mbt \| \| POLO \| \| CG1227 \| \|  \| \|  \| \| VRK \| \| wun2 \| \|  \| \| PP2C \| \| hppy \| \| pll \| \| wdb \| \|  \| \| Pka-C1 \| \| SIK SIK2 \| \|  \| \| Drl-2 \| \| Sdr \| \| Pp2c1 \| \| dco \| \| alph \| \| CAMKIIB \| \| Nek2 \| \| HIPK \| \| hop \| \| SIK3 \| \|  \| \| Tao-1 \| \|  \| \|  \| \| Eip63E \| \|  \| |

Common modifiers in *en > lgli* (or *en-GAL4*) and *GMR-GAL4* and *GMR > aPKC^CA^* and/or *GMR > crb^intra^* (Class 4).

| RNAi line | CG | Gene |
| --- | --- | --- |
| \| 101624  6229 \| \| --- \| \| 40743 \| \| 104860 \| \| 9156-R1 (II)  107071 37279 \| \|  \| \| 100708 \| \| 27785 \| \| 105525 \| \| 106130 41838 \| \| 43783 \| \| 105732 46425 \| \| 51227 \| \| 2055R-1 \| \| 13664 \| \| 991 \| \| 41134 41136 \| \| 16973R-1 (III) \| \| 39857 \| \| 106174 \| \| 106497 \| \| 104169 \| \| 100163 42947 \| \| 38319 \| \| 105353 976 \| \| 107158 49558 \| \| 25317 \| \| 103976 \| \| 106928 \| \| 101997 \| | \| CG9985 \| \| --- \| \| CG9493 \| \| CG9311 \| \| CG9156 \| \| CG7904 \| \| CG7873 \| \| CG7004 \| \| CG6593 \| \| CG5363 \| \| CG4527 \| \| CG34412 \| \| CG2252 \| \| CG2049 \| \| CG2028 \| \| CG18402 \| \| CG1725 \| \| CG16973 \| \| CG14992 \| \| CG12072 \| \| CG11486 \| \| CG11228 \| \| CG11221 \| \| CG10033 \| \| CG8222 \| \| CG5169 \| \| CG32505 \| \| CG1609 \| \| CG7177 \| \| CG12217 \| | \| sktl \| \| --- \| \| Pez \| \|  \| \| PP1-13C \| \| put \| \| Src42A \| \| fwd \| \| Pp1 \| \| cdc2 \| \| slik \| \| tlk \| \| fs(h) \| \| PRK2 \| \| Ckl \| \| InR \| \| dlg \| \| msn \| \| Ack \| \| wts \| \|  \| \| hpo \| \| PKN \| \| DG2, for \| \| Pvr \| \| Gckiii \| \| PP4-19C \| \| Gcn2 \| \| Wnk \| \| PpV \| |
